# Neural defects caused by total and Wnt1-Cre mediated ablation of p120ctn in mice

**DOI:** 10.1186/s12861-020-00222-4

**Published:** 2020-08-03

**Authors:** Tim Pieters, Ellen Sanders, Huiyu Tian, Jolanda van Hengel, Frans van Roy

**Affiliations:** 1 Molecular Cell Biology Unit, Center for Inflammation Research, VIB, Technologiepark 71, B-9052 Ghent, Belgium; 2 Department of Biomedical Molecular Biology, Ghent University, Technologiepark 71, B-9052 Ghent, Belgium; 3 Present address: Faculty of Medicine and Health Sciences, Ghent University Hospital, Corneel Heymanslaan 10, 9000 Ghent, Belgium; 4 Present address: Ministry of Education, College of Life Sciences, Shandong University, Jinan, People’s Republic of China

**Keywords:** p120 catenin, Knockout mouse, Wnt1, NTD, Exencephaly, Neural fold closure, Cadherins, Actin binding proteins

## Abstract

**Background:**

p120 catenin (p120ctn) is an important component in the cadherin-catenin cell adhesion complex because it stabilizes cadherin-mediated intercellular junctions. Outside these junctions, p120ctn is actively involved in the regulation of small GTPases of the Rho family, in actomyosin dynamics and in transcription regulation. We and others reported that loss of p120ctn in mouse embryos results in an embryonic lethal phenotype, but the exact developmental role of p120ctn during brain formation has not been reported.

**Results:**

We combined floxed p120ctn mice with Del-Cre or Wnt1-Cre mice to deplete p120ctn from either all cells or specific brain and neural crest cells. Complete loss of p120ctn in mid-gestation embryos resulted in an aberrant morphology, including growth retardation, failure to switch from lordotic to fetal posture, and defective neural tube formation and neurogenesis. By expressing a wild-type p120ctn from the ROSA26 locus in p120ctn-null mouse embryonic stem cells, we could partially rescue neurogenesis. To further investigate the developmental role of p120ctn in neural tube formation, we generated conditional p120ctn^fl/fl^;Wnt1Cre knockout mice. p120ctn deletion in Wnt1-expressing cells resulted in neural tube closure defects (NTDs) and craniofacial abnormalities. These defects could not be correlated with misregulation of brain marker genes or cell proliferation. In contrast, we found that p120ctn is required for proper expression of the cell adhesion components N-cadherin, E-cadherin and β-catenin, and of actin-binding proteins cortactin and Shroom3 at the apical side of neural folds. This region is of critical importance for closure of neural folds. Surprisingly, the lateral side of mutant neural folds showed loss of p120ctn, but not of N-cadherin, β-catenin or cortactin.

**Conclusions:**

These results indicate that p120ctn is required for neurogenesis and neurulation. Elimination of p120ctn in cells expressing Wnt1 affects neural tube closure by hampering correct formation of specific adhesion and actomyosin complexes at the apical side of neural folds. Collectively, our results demonstrate the crucial role of p120ctn during brain morphogenesis.

## Background

During cranial neurulation in mammals, the neuroepithelium differentiates from a dorsal midline ectoderm into a neural plate. The edges of the neural plate then elevate to form neural folds, which later fuse to form the neural tube. In the mouse head, fusion is initiated at the boundary between the hindbrain and the cervical region [1, 2]. Two more initiation sites are located at the forebrain-midbrain boundary and at the rostral extremity of the forebrain. These closures extend cephalically and caudally to finish the final fusion of the cranial neural tube, which is the embryonic precursor of the brain and spinal cord. Failure to complete the neurulation process leaves the neural tube open; these abnormalities are known as neural tube closure defects (NTDs) [3]. NTDs are a common birth defect in humans and occur in about 1 per 500 live births. Defects in posterior neuropore closure result in spina bifida, whereas failure of anterior neuropore closure often results either in exencephaly (protrusion of an excessive amount of brain outside of the skull) or in anencephaly (absence of (fore)brain), frequently leading to embryonic death [4]. In mouse, more than 240 mutations are reportedly related to NTDs, and this number is further increasing [5, 6].

Adherens junctions play a crucial role in the morphogenetic processes controlling neural tube closure as well as neural crest (NC) cell migration and formation of skeletal structures [7, 8]. Adherens junctions are cell–cell junctions composed of several classes of proteins, including cadherins and catenins. Cadherins (e.g. E- and N-cadherin) are important calcium-dependent cell–cell adhesion molecules. Classic cadherins have a single transmembrane domain and a cytoplasmic domain that associates with members of the armadillo protein family, such as β-catenin and p120ctn. Those catenins have important roles in developmental signaling pathways [9–12]. Some parallels exist between the structures and functions (cadherin association and gene control) of the armadillo proteins β-catenin and p120ctn. Unlike p120ctn, which binds to a juxtamembrane domain of classic cadherins, β-catenin binds to a C-terminal domain in these cadherins [13]. p120ctn is an essential multifunctional protein that contributes to stabilization of cadherins by inhibiting their degradation [14, 15]. It also regulates gene expression in the nucleus by removing the transcriptional repressor Kaiso [16, 17]. In addition, p120ctn modulates the activities of small GTPases of the Rho family, which control the dynamics of the cytoskeleton and the assembly of adherens junctions [18–20].

Most members of the cadherin-catenin complex are essential for mouse embryonic development [21–23]. The phenotypes of E- and N-cadherin deficient embryos are in line with their temporal expression pattern [24]. E-cadherin is the first cadherin molecule that is expressed during development, is upregulated during compaction of morulas, and is required for trophectoderm formation in the blastocyst [21]. As the neural tube forms, the neuroectoderm gradually loses E-cadherin and gains N-cadherin expression instead [8, 25]. This switch from E-cadherin to N-cadherin coincides with a cellular transformation of immotile epithelial cells to motile mesenchymal cells, a process which is called epithelial-to-mesenchymal transition (EMT). EMT processes are required for normal neural tube morphogenesis and for neural crest cell development and behavior [26]. Indeed, newly expressed N-cadherin appears in the nascent mesoderm that migrates from the primitive streak during gastrulation, and complete ablation of N-cadherin induces malformation of the heart and neural structures [22].

Loss of the catenin molecules, β-catenin and p120ctn, also results in defects during gastrulation [23, 27, 28]. While we reported that early stages of gastrulation occur normally in absence of p120ctn [29], Hernandez-Martinez and colleagues reported defects in the migration of mesoderm and bifurcation of the posterior axis in p120ctn-null mouse embryos [28]. Nevertheless, the exact in vivo roles of mouse p120ctn in neurulation, neurogenesis, and formation of head skeletal structures remain elusive. To investigate these roles, we totally or conditionally inactivated p120ctn in developing embryos. We show that complete p120ctn ablation resulted in aberrant embryonic morphology, failure to switch from lordotic to fetal posture, defective neural tube formation and neurogenesis in mid-gestation embryos. Conditional ablation of p120ctn in Wnt1-expressing cells often resulted in exencephaly. N-cadherin, E-cadherin, β-catenin and the actin-binding protein cortactin were found to be downregulated together with p120ctn ablation in a very focal region at the top edges of neural folds, although not at the lateral sites of these p120ctn lacking neural folds. We also found that p120ctn stabilized the actin-binding protein Shroom3 at the neural folds. Collectively, our results indicate that p120ctn plays a crucial role in neurulation, as its ablation results in defective neural tube closure and generation of NTDs.

## Results

### p120ctn is required for turning of the mouse embryo and neurulation

**Fig. 1:**
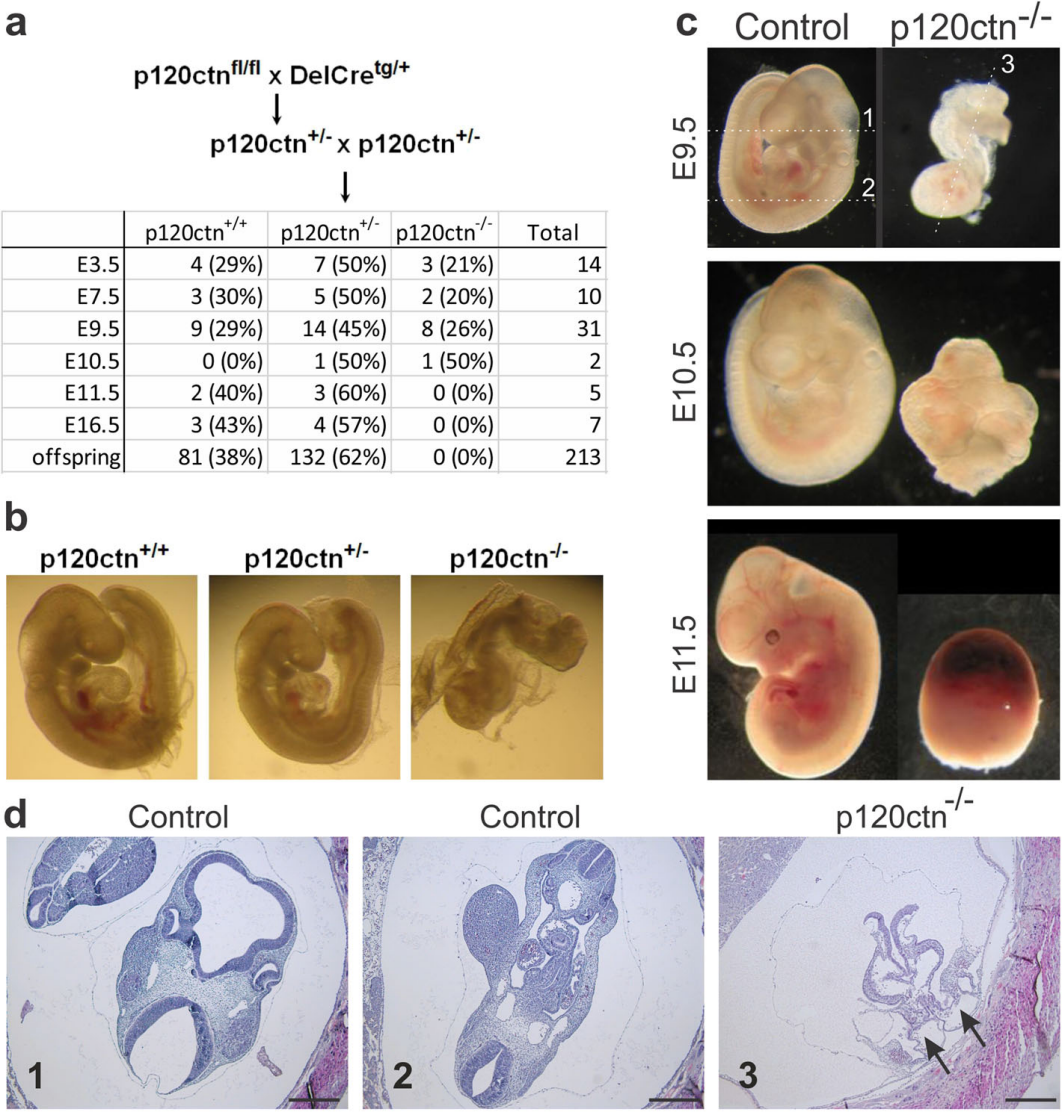
Impaired embryonic turning and neural tube closure defect upon p120ctn loss in mouse. **a** Breeding scheme to obtain p120ctn^−/−^ embryos in timed matings. The table depicts numbers and percentages of p120ctn^+/+^, p120ctn^+/−^ and p120ctn^−/−^ embryos that were recovered at the developmental stages indicated (E: embryonic day). **b** p120ctn^+/+^, p120ctn^+/−^ and p120ctn^−/−^ littermates at E9.5. **c** Control (p120ctn^fl/fl^) and p120ctn^−/−^ embryos were collected at different developmental stages as indicated. From E11.5 on, all p120ctn^−/−^ embryos were resorbed. **d** H&E stained paraffin sections of control and p120ctn^−/−^ E9.5 embryos. The section planes #1 to 3 are indicated in panel **c** as dashed lines: #1 and 2 in the E9.5 control embryo, and #3 in the p120ctn^−/−^ embryo. Two arrows point at the posterior axis duplication in the p120ctn^−/−^ embryo. Scale bars: 200 μm

Mice lacking p120ctn have a defective gastrulation and die around mid-gestation [11, 28, 30], but the exact developmental defects beyond the gastrulation stage have not been described in detail. We performed timed matings for generation of p120ctn full KO embryos (Fig. 1a). Recently, we showed that upon genetic ablation of p120ctn preimplantation development and early stages of gastrulation occur normally [29]. However, from mid-gestation (E9.5) on, p120ctn-null embryos exhibited an aberrant morphology, including growth retardation and failure to switch from lordotic to fetal posture, a process which is called ‘turning’ (Fig. 1b). In E9.5 control embryos the neural tube is normally closed, whereas E9.5 p120ctn-null embryos displayed a strikingly defective neural tube formation (Fig. 1c,d). At this stage and in line with a recent report [28], p120ctn-null embryos also displayed posterior axis duplication (Fig. 1d, arrows). Growth retardation and developmental defects progressed over time and from E11.5 onwards, all p120ctn-null embryos were resorbed (Fig. 1a,c). In conclusion, mouse p120ctn is important for correct neurulation in vivo.

### p120ctn is essential for neurogenesis in mESCs and E9.5 embryos

**Fig. 2:**
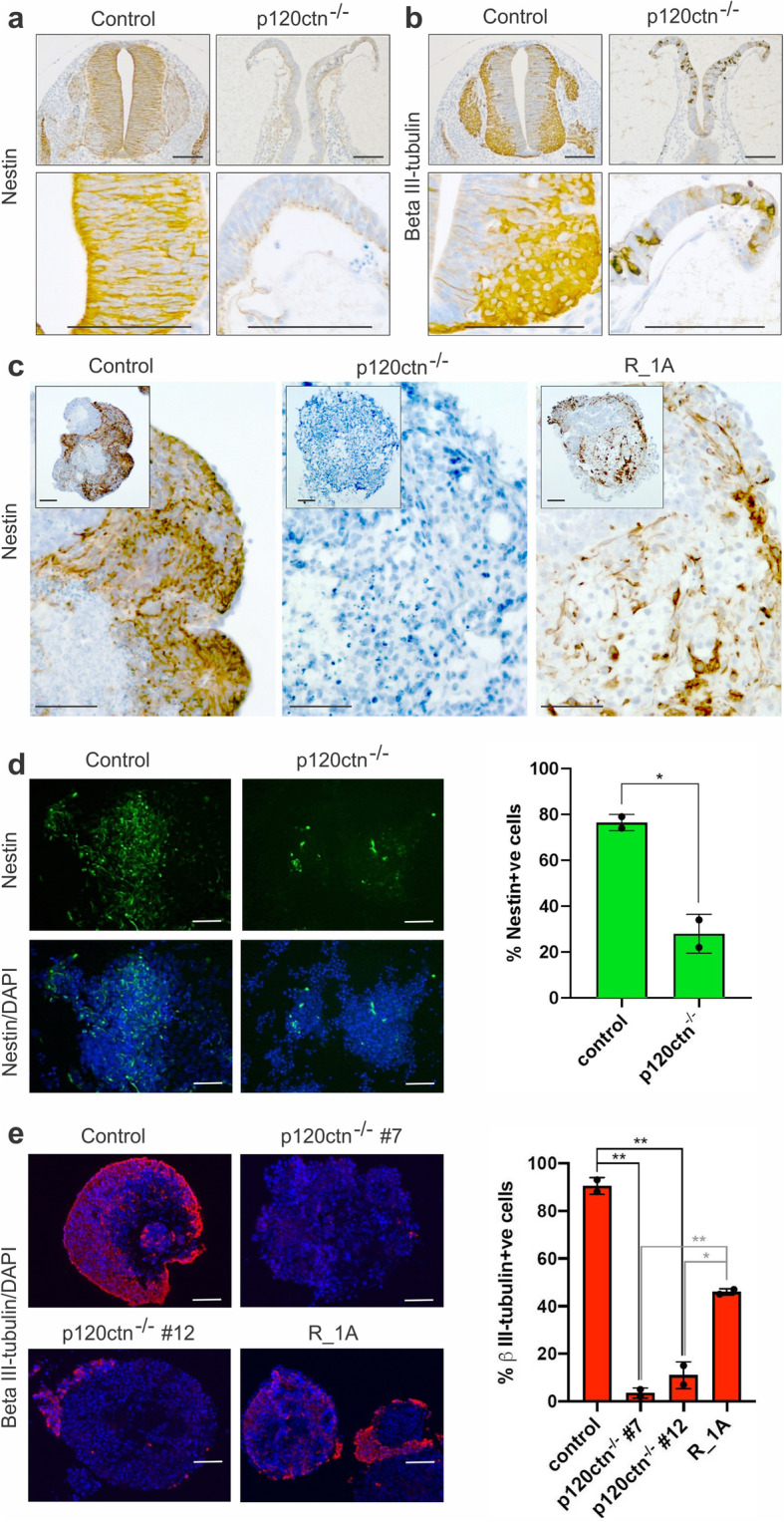
Reduced neurogenesis in p120ctn-null embryos and mESCs. **a**, **b** Immunohistochemical detection of Nestin (**a**, neuronal progenitor marker) and βIII-tubulin (**b**, neuronal marker) on paraffin sections from E9.5 control (p120ctn^fl/fl^ littermates) and p120ctn-null embryos (three embryos per setup). Scale bars: 100 μm. **c** Immunohistochemical detection of Nestin in 30-day old EBs generated from control and p120ctn-null mESCs, and from an RMCE-targeted [29] mESC line expressing R26-driven wild-type p120ctn isoform 1A (designated R_1A). Scale bars: 100 μm. **d** Immunostaining of Nestin (left panel) in control and p120ctn-depleted mESCs, which were induced towards neurectoderm differentiation by culturing for 6 d in N2B27 medium. p120ctn loss abrogated mESC differentiation towards the neuronal fate. This neural induction experiment was performed twice. Scale bars: 25 μm. Graph (right panel) depicting the percentage of Nestin-positive cells in control and p120ctn-null cultures. The error bar represents the standard deviation of two technical replicates (coverslips). The *P*-value (* 0.0175) was calculated via an unpaired t-test. **e** Immunostaining of βIII-tubulin (left panel) in control and p120ctn-null mESCs that were converted into embryoid bodies for 4 d, then treated with retinoic acid for 4 d, and finally cultured for 8 d in N2B27 medium. We analysed a control mESC line, two independent p120ctn-null mESCs lines (#7 and #12) and rescued R_1A mESCs. Scale bars: 50 μm. Graph (right) depicting the percentage of βIII-tubulin-positive cells. The error bar represents the standard deviation of two independent quantifications in which at least 500 cells were scored per condition. The *P*-values (black: ** 0.0011; ** 0.0024; grey: ** 0.0018; * 0.0136) were calculated via an unpaired t-test

Next, we wondered whether also neurogenesis was affected in the absence of p120ctn. In E9.5 control embryos, emerging neurons can be identified in their neural tubes by use of neuronal markers such as Nestin and βIII-tubulin (Fig. 2a,b). In contrast, in E9.5 p120ctn-null embryos neural tube formation was aberrant and there was reduced expression of neuronal markers (Fig. 2a,b). On the other hand, expression patterns of mesodermal markers such as smooth muscle actin (SMA) and troponin-T were not affected by the p120ctn ablation (Fig. S1a,b).

To exclude that the lack of emerging neurons in p120ctn-null embryos is due to a developmental delay rather than to the essential need for p120ctn to induce neurogenesis, we performed in vitro neural differentiation experiments in control and p120ctn-null mESCs. We previously reported on the derivation of control and p120ctn-null mESCs [29]. We used such mESCs now to induce either spontaneous differentiation or neural-directed differentiation by using two protocols. Spontaneous differentiation in embryoid bodies (EBs) was analyzed by culturing control and p120ctn-null mESCs for 30 days onto low-adherent bacterial-grade plates, followed by immunohistochemical analysis. Whereas control EBs contained lots of Nestin-positive cells, these were clearly absent in p120ctn-null EBs (Fig. 2c). In the first neurogenesis protocol [31], we cultured control and p120ctn-null mESCs for 6 days in N2B27 medium and found that p120ctn-depleted cells produced a significantly lower number of Nestin-positive neural progenitors compared to control mESCs (Fig. 2d). In a second protocol [32], embryoid bodies (EBs) were made of control and p120ctn-null mESCs and subsequently cultured in N2B27 medium with or without retinoic acid (see details in the Methods section). Similar to the results of the previous approach, a significant reduction of βIII-tubulin positive cells could be identified for two independent p120ctn-null mESCs clones (Fig. 2e). To test the neuronal differentiation potential in vivo, we subcutaneously injected control and p120ctn-null mESCs into athymic nude mice and monitored teratoma formation. Both control and p120ctn-null mESCs formed teratomas although at low efficiency. Only control but not p120-null teratomas were positive for neuronal markers Nestin and βIII-tubulin (Fig. S2). Finally, we performed a rescue experiment [29], using RMCE-targeted p120ctn-null mESCs expressing R26-driven wild-type p120ctn isoform 1A (designated R_1A). We found that neurogenesis was partially restored in R_1A EBs (Fig. 2c,e). These data support an important role for p120ctn, both in vitro and in vivo, in instructing cells to commit to the neurectoderm lineage.

### Neural tube defects (NTD) upon p120ctn ablation at sites of Wnt1-Cre expression

To analyze the role of p120ctn during neurulation while avoiding developmental defects in p120ctn-null embryos, we generated mice in which p120ctn deletion was restricted to Wnt1-expressing cells. Wnt1-Cre mediated KO of p120ctn was obtained by breeding mice that had inherited a floxed p120ctn allele [11] with Wnt1-Cre transgenic mice [33]. Cre-negative p120ctn^fl/fl^ littermates were used as controls in all experiments described below. The Wnt1-driven Cre recombinase is expressed in the cranial neural plate, the dorsal neural tube and in all neural crest cells within the early embryo [33, 34]. We have confirmed this expression pattern by performing timed matings with Wnt1-Cre and R26-*LacZ* reporter mice [35], followed by X-gal-staining of the embryos. Expression was found at the reported sites from the 6-somite stage (about E8.0) on (Fig. S3), but was not observed at earlier stages.

**Fig. 3:**
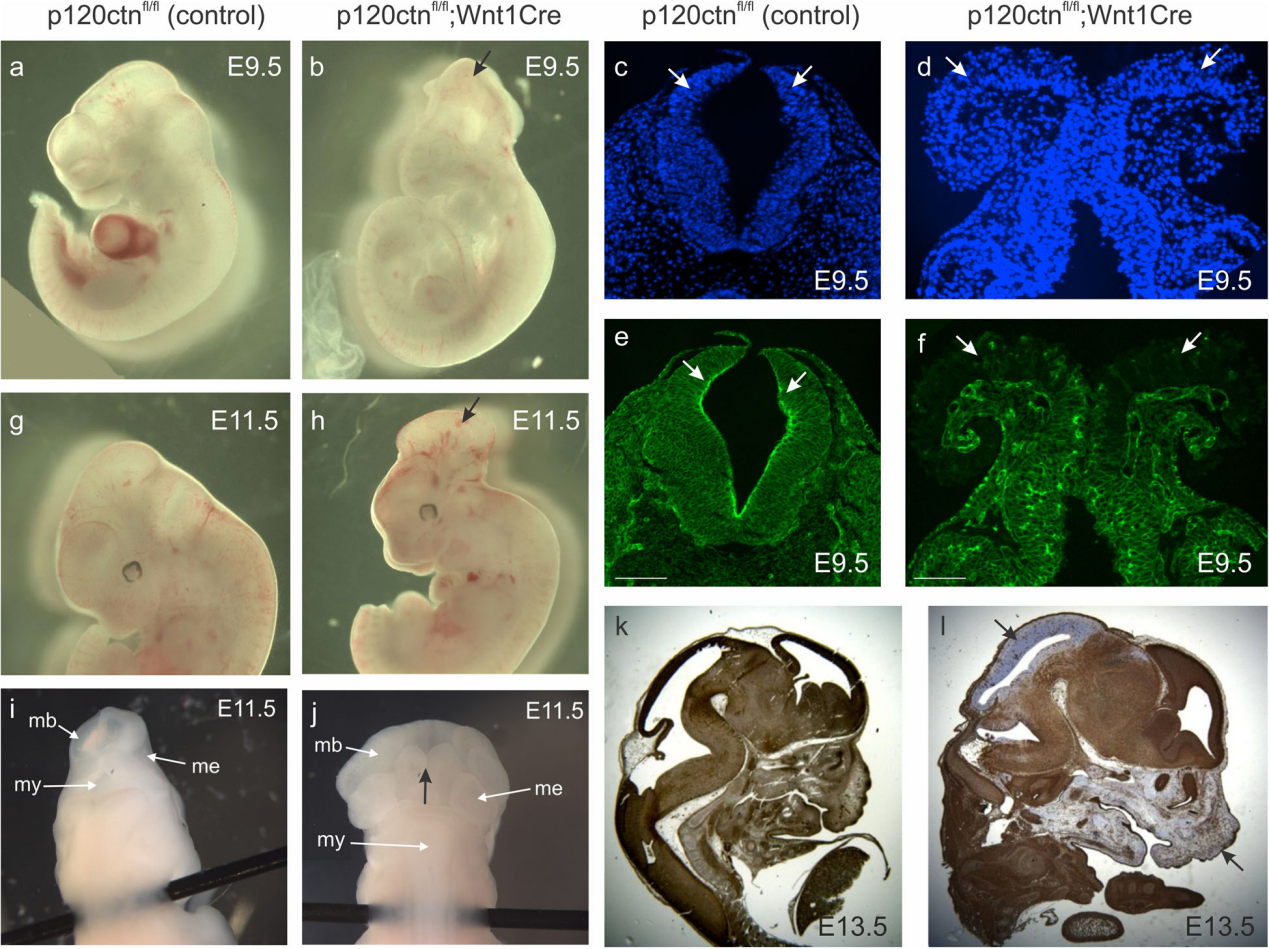
Conditional ablation of the mouse p120ctn encoding gene by the action of Wnt1-Cre results in neural tube defects (NTDs). Control mice carried two floxed p120ctn encoding alleles (p120ctn^fl/fl^) but lacked the Wnt1-Cre gene; mutant p120ctn^fl/fl^;Wnt1Cre mice had ablated p120ctn encoding alleles at sites of Wnt1Cre expression. **a**, **b** Lateral views at E9.5 of a control embryo (**a**), and a mutant embyo, the latter displaying a NTD (**b**, arrow). **c-f** Coronal sections at the level of the hindbrain of an E9.5 control mouse (**c**, **e**) and an E9.5 mutant mouse (**d**, **f**) were stained with either DAPI (**c**, **d**) or with anti-p120ctn antibody (**e**, **f**). Arrows in (**c**, **e**) point at normal, almost closed p120ctn-positive neural folds. Arrows in (**d**, **f**) point at extended unfolded p120ctn-lacking neural folds. Scale bars: 20 μm. **g**-**j** External lateral (**g**, **h**) and dorsal (**I**, **j**) views of E11.5 head structures. Control embryos (**g**, **i**) show normal brain development (mb, midbrain; me, metencephalon; my, myelencephalon). Mutant embryos show exencephaly (**h**, arrow), expanded mb and me structures and non-closure of the neural tube (**j**, black arrow). **k**, **l** IHC of p120ctn in sagittal sections of the head region of E13.5 embryos. There is ubiquitous expression in the control embryo (**k**), but an obvious lack of p120ctn expression in the enlarged hindbrain region and the facial structures of the mutant embryo (**l**, arrows)

We initially analyzed 42 mutant mice with a p120ctn^fl/fl^;Wnt1Cre genotype and a mixed C57BL/6 and FVB/N background. Of these offspring embryos, 29 (69%) survived after birth and showed only minor brain malformations and craniofacial abnormalities, including a small elevation of the midbrain with respect to the skull and a larger space between the frontal bones. This was determined by E. Descamps and D. Adriaens (Ghent University) using orthogonal-plane fluorescence optical sectioning (OPFOS) microscopy [36, 37]. In addition, these survivors displayed defects in the eyes [38]. The remaining 13 (31%) p120ctn^fl/fl^;Wnt1Cre embryos died prenatally and showed various degrees of NTDs (Fig. 3). Abnormalities in the neural tube of p120ctn^fl/fl^;Wnt1Cre embryos were obvious from E9.5 on (Fig. 3a,b), the time at which the neural tube normally closes, and were similar to these of the full p120ctn KO at E9.5 (Fig. 1c,d). The young p120ctn^fl/fl^;Wnt1Cre embryos showed a defect in anterior neural tube closure at the level of the mid- and hindbrain. Loss of p120ctn was confirmed on sections of the hindbrain of E9.5 embryos, where the strong p120ctn expression at the apical side of control neural folds (Fig. 3c,e; arrows), was convincingly ablated in the abnormal out-folding structures of the p120ctn^fl/fl^;Wnt1Cre brains (Fig. 3d,f; arrows). This indicates that p120ctn is required in Wnt1 expressing cells of the brain to allow proper neural tube closure.

At E11.5, the neural tube was fully closed in control embryos (Fig. 3g,i), but still open in the p120ctn^fl/fl^;Wnt1Cre embryos with NTDs (Fig. 3h,j). In addition, the mutant mice suffered from exencephaly: lateral extensions of the neural folds and a protruding extra brain mass (Fig. 3j). In E13.5 p120ctn^fl/fl^;Wnt1Cre embryos, p120ctn was strikingly reduced in the hindbrain and facial structures, in comparison to control embryos (Fig. 3k,l; arrows), what is in concordance with the expression pattern of Wnt1-driven Cre (Fig. S3). The NTDs did not ameliorate over time and remained obvious as late as E16.5. Eventually, embryos with such severe phenotype died before E17.5. At that time, dramatic defects in frontal, parietal and interparietal skull bones were observed [37].

When we backcrossed p120ctn^fl/fl^;Wnt1Cre mutant mice to the C57BL/6 background for 10 times, the NTD frequency progressively decreased to 1%, while total counts for offspring on a mixed background revealed 18% embryos displaying exencephaly (35 out of 191 p120ctn^fl/fl^;Wnt1Cre^+^ embryos analyzed). This indicates that unknown strain-dependent genetic factors modulate the final effects of loss of p120ctn on NTDs.

### Analysis of early midbrain, hindbrain and proliferation markers in p120ctn^fl/fl^;Wnt1Cre embryos

**Fig. 4:**
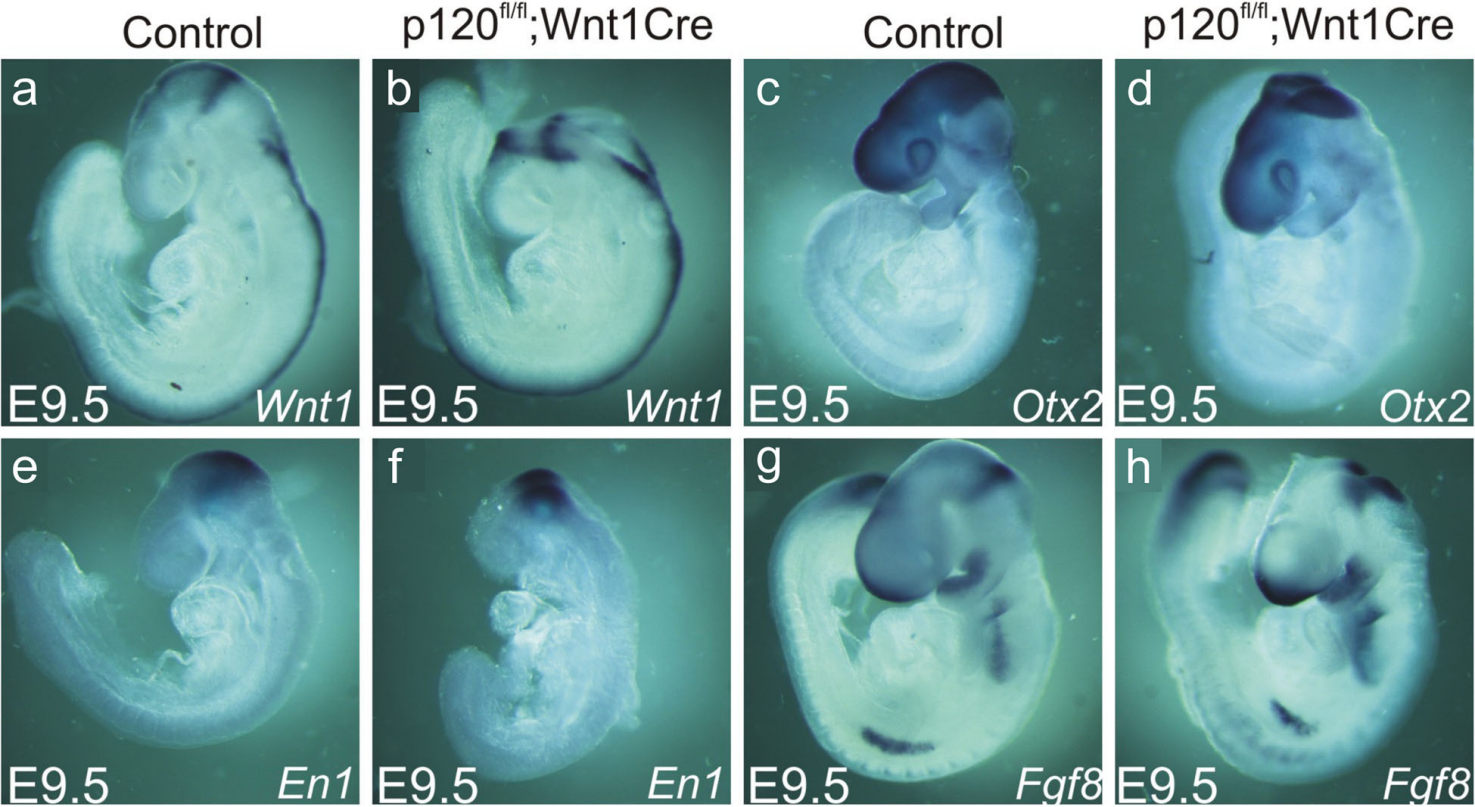
Whole-mount in situ hybridization of representative brain markers in control and p120ctn^fl/fl^;Wnt1Cre mouse embryos at E9.5. The analysis revealed no expression difference between these two genotypes. Riboprobes were used for *Wnt1* (**a**, **b**), *Otx2* (**c**, **d**)*, En1* (**e**, **f**)*,* and *Fgf8* (**g**, **h**)

To analyze whether the NTDs in p120ctn^fl/fl^;Wnt1Cre embryos were due to a major dysregulation of the gene networks regulating brain development, we examined representative midbrain and hindbrain markers (*Wnt1, Otx2, En1* and *Fgf8*) by whole-mount in situ hybridization on mutant and control embryos at E9.5 (Fig. 4). The expression patterns in control embryos (Fig. 4a, c, e, g) was fully in accordance with published studies [39–42]. We observed the same expression patterns in p120ctn^fl/fl^;Wnt1Cre embryos, even in the neural tube opening region of mutant embryos displaying severe NTD (Fig. 4b, d, f, h).

**Fig. 5:**
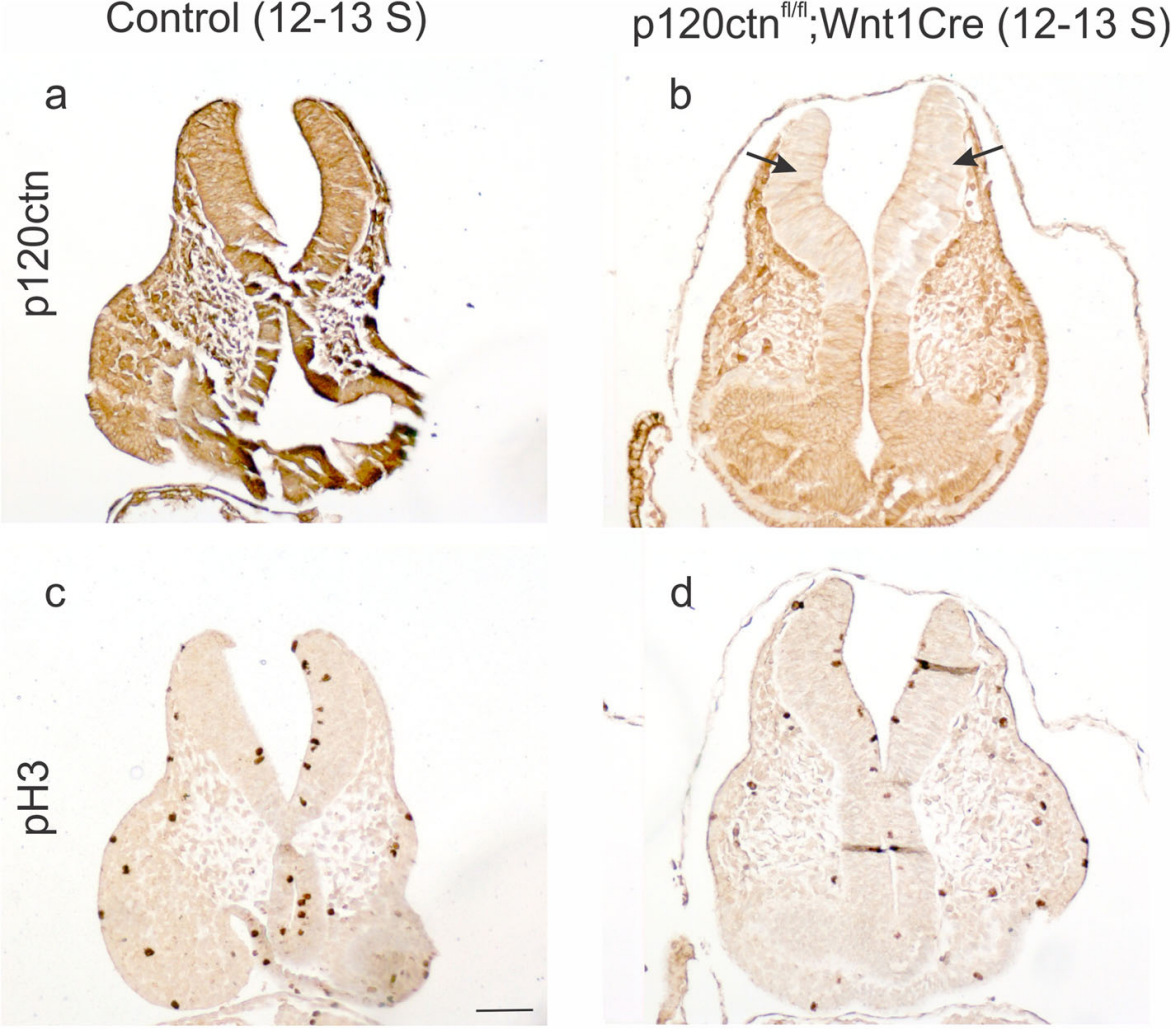
No prominent cell proliferation changes in p120^fl/fl^;Wnt1Cre mutant mice. Sections through the hindbrain region of 12–13 somite embryos are shown. **a**, **b** Expression of p120ctn was prominent in the neural folds of a control embryo (**a**), but ablated in the neural folds of a mutant embryo (**b**, arrows). **c**, **d** Immunodetection of phosphorylated Histone 3 (pH 3) showed similar activity in control and mutant embryos. Scale bar: 20 μm

Next, we addressed cell proliferation activity by IHC for phopho-Histone 3 (pH 3) in control and p120ctn^fl/fl^;Wnt1Cre embryos. Histone H3 is specifically phosphorylated in late G2 and mitosis. Regions of p120ctn ablation were revealed by IHC staining on consecutive paraffin sections. Staining of embryos with 12–13 somites (about E8.5) showed that p120ctn ablation in the elevated neural folds was not correlated with drastically changed pH 3 activity (Fig. 5). For older embryos (18–22 somites; about E9.0–9.5), we compared sections of control mice, mutant mice with closed neural tube and mutant mice with unfolded extended neural folds (Fig. S4). Similar activity of pH 3 was seen at the apical side of control neural tube, mutant neural tube and mutant unfolded neural folds (Fig. S4d-f).

### Abnormal N-cadherin expression in brains of p120ctn^fl/fl^;Wnt1Cre embryos

**Fig. 6:**
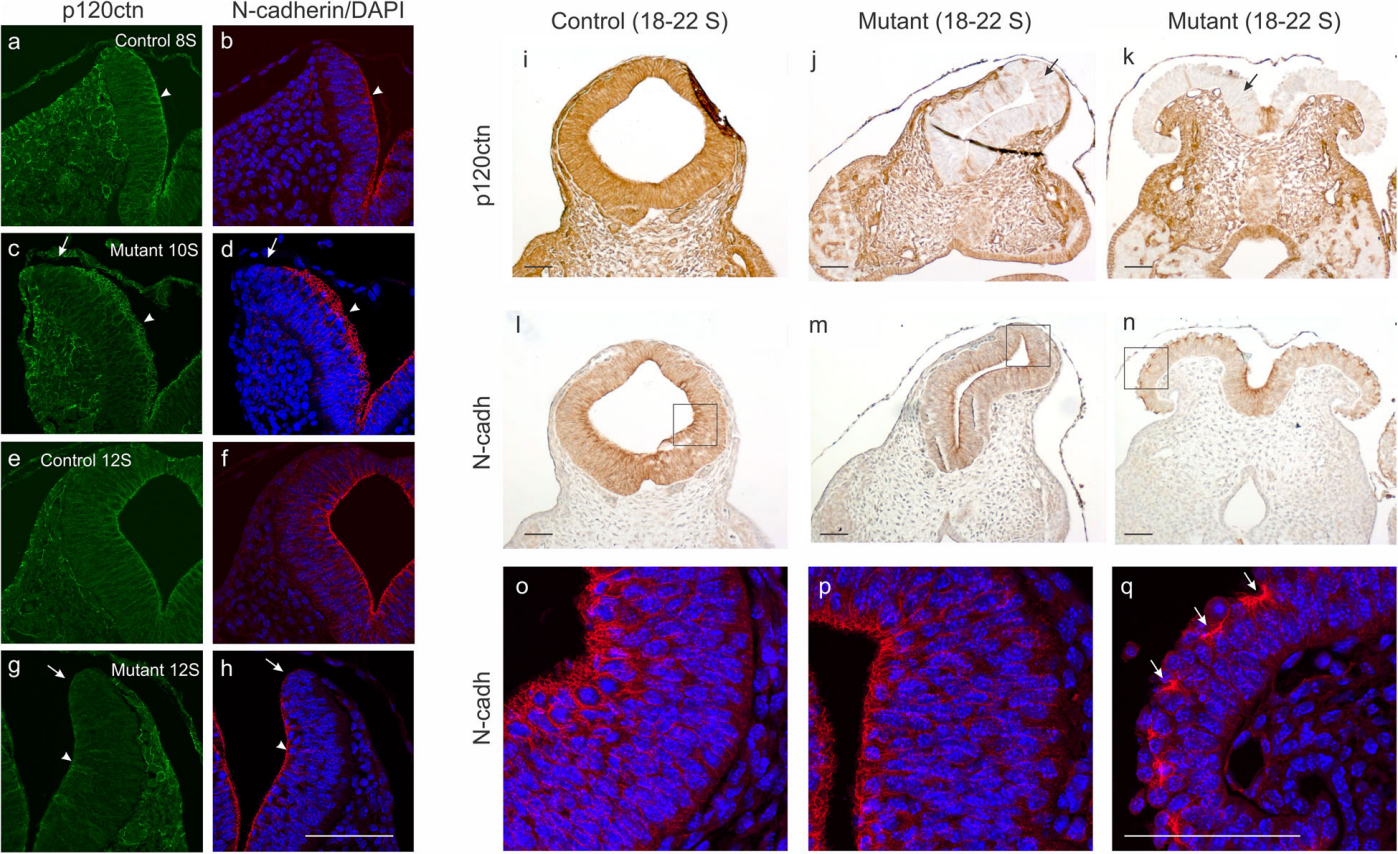
Focal abnormalities of N-cadherin expression in the neural folds of p120fl/fl;Wnt1Cre mutant mice. **a**-**h** Immunofluorescence of p120ctn (left panels) and N-cadherin (right panels; nuclear counterstaining by DAPI) on neural folds/tube in the hindbrain region of young embryos (lateral sides of folds are annotated by arrowheads). Detection was on 8-somite (**a**, **b**) and 12-somite (**e**, **f**) control embryos, and on 10-somite (**c**, **d**) and 12-somite (**g**, **h**) mutant embryos. The mutant embryos showed focal loss of both p120ctn and N-cadherin at the very tip of the neural folds (arrows in **c**, **d**, **g**, **h**), but in the 12-somite mutant embryo the p120ctn-lacking lateral side of the neural folds retained strong N-cadherin positivity (arrowheads in **g**, **h**). Scale bar: 20 μm. **i**-**q** IHC of p120ctn (**i**-**k**) and of N-cadherin (**l**-**n**) on neural folds/tube in the hindbrain region of 18–22 somite embryos. Scale bars: 20 μm. **o**-**q** Immunofluorescence of N-cadherin in areas, corresponding to the squares in panels **l**-**n**. The embryos analysed were control (**i**, **l**, **o**), mutant with closed neural tube (**j**, **m**, **p**) and mutant with NTD (**k**, **n**, **q**). p120ctn-lacking neural folds/tube (arrows in **j**, **k**) retained N-cadherin positivity (**m**, **n**, **p**, **q**) although N-cadherin was locally aggregated in case of NTD (**n**, arrows in **q**)

Since p120ctn stabilizes expression of classic cadherins, including E- and N-cadherin [14, 15], loss of N-cadherin might explain the NTDs observed in p120ctn^fl/fl^;Wnt1Cre embryos. To scrutinize the mechanism underlying the NTD phenotype we investigated by double immunofluorescence the co-expression of N-cadherin and p120ctn in the brains of control and mutant embryos at stages 8S -12S (Fig. 6). In 10-somite mutant embryos, p120ctn expression was locally affected at the top of the neural fold (Fig. 6c, arrow), and this corresponded to local loss of N-cadherin (Fig. 6d, arrow). At the 12-somite stage of the control embryo, expression of p120ctn and N-cadherin nicely colocalized at the apical side of the closed neural tube (Fig. 6e,f). Again, the top of the neural fold in the mutant embryo showed loss of both p120ctn and N-cadherin expression (Fig. 6g,h; arrows). Surprisingly, at the lower levels of this neural fold, p120ctn expression was largely gone, while N-cadherin was still strongly expressed (Fig. 6g,h; arrowheads). At a later stage (18-22S), we compared a control embryo with p120ctn^fl/fl^;Wnt1Cre (mutant) embryos that showed either normal neural tube closure or opened neural folds (Fig. 6i-q). Despite complete p120ctn ablation in the closed neural tube of one KO embryo (Fig. 6j), N-cadherin expression seemed to be normal (Fig. 6m,p). In contrast, in case of the extended neural folds with completely ablated p120ctn expression in the other mutant embryo (Fig. 6k), N-cadherin was also expressed but appeared to be aggregated (Fig. 6n,q). Also in E9.5 mutant embryos (25-30S), N-cadherin was apparently strongly expressed in p120ctn-ablated extended neural folds, both at the well-organized basal side, and at the disorganized and less cohesive apical side (arrows in Fig. S5).

### Abnormal E-cadherin expression in brains of p120ctn^fl/fl^;Wnt1Cre embryos

**Fig. 7:**
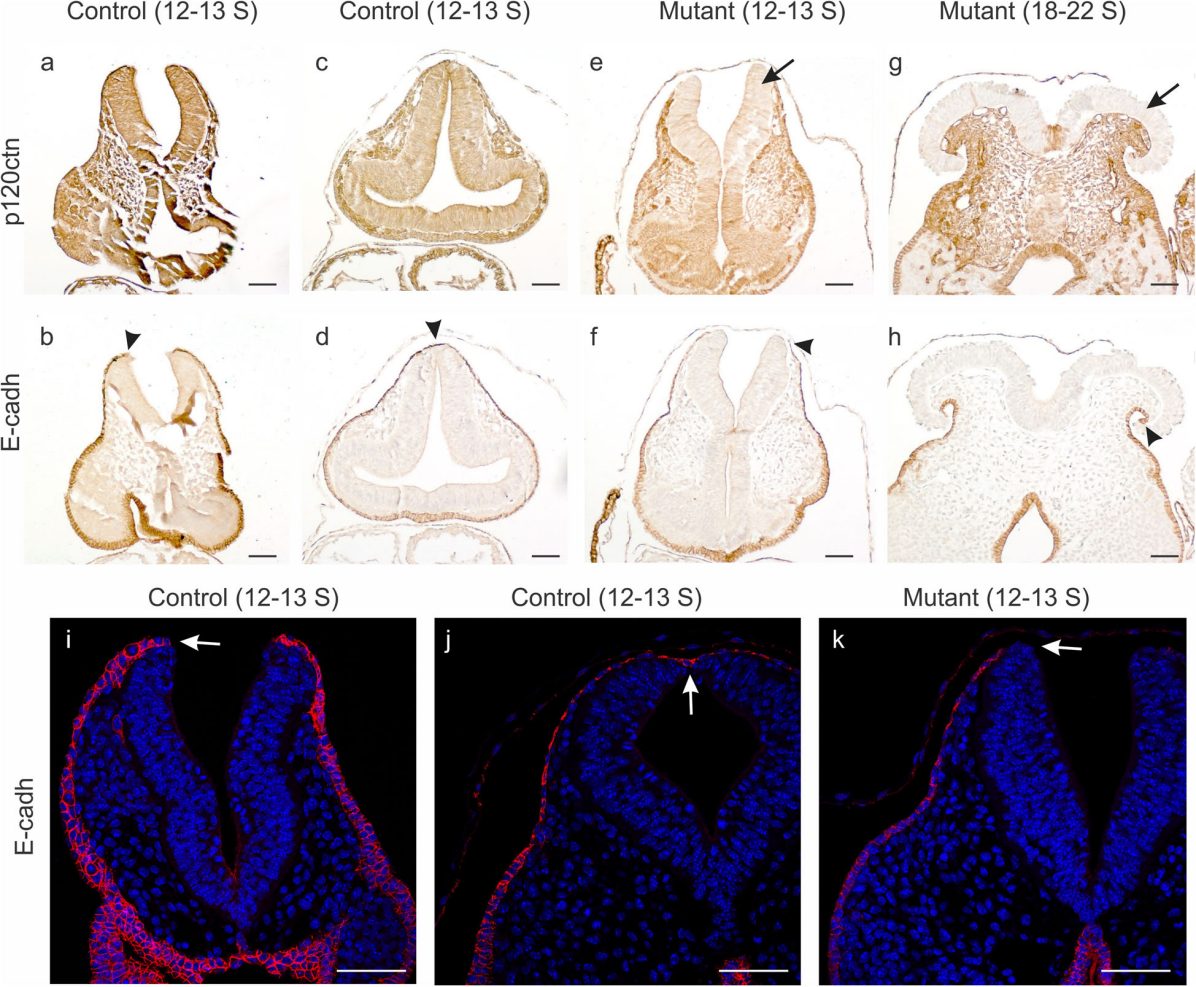
Focal abnormalities of E-cadherin expression in non-neural ectoderm of p120fl/fl;Wnt1Cre mutant mice. **a**-**h** IHC of p120ctn (top panels) and E-cadherin (bottom panels) on neural folds/tube in the hindbrain region of control and mutant embryos. Detection was on a 12–13 somite control embryo with open neural folds (**a**, **b**), on a 12–13 somite control embryo with closed neural tube (**c**, **d**), on a 12–13 somite mutant embryo with open neural folds (**e**, **f**), and on an 18–22 somite mutant embryo with NTD (**g**, **h**). The mutant embryos showed the expected loss of p120ctn (arrows in **e**, **g**). In **b**-**h**, the respective ends of the E-cadherin-positive non-neural ectoderm flanking the neural folds/tube are indicated by arrowheads. **i**-**k** Immunofluorescence of E-cadherin (nuclear counterstaining by DAPI) in the hindbrain region of either 12–13 somite control embryos with open neural folds (**i**) or a closed neural tube (**j**), or a 12–13 somite mutant embryo with open neural folds (**k**). Arrows indicate the very tip of the neural fold (**i**, **k**) or the dorsal closure point of the neural tube (**j**). There is an E-cadherin deficiency at the very tip of the neural folds of the mutant embryo (**k**, arrow). Scale bars: 20 μm

Neurogenesis is characterized by cadherin switching [25]: the epithelial E-cadherin is downregulated and replaced by expression of N-cadherin. The E- to N-cadherin switch was not affected in the p120ctn ablated neuro-epithelial regions of p120ctn^fl/fl^;Wnt1Cre mice (see above). During normal neurulation, a non-neural ectodermal layer apposing the rising neural folds continues to express E-cadherin. In view of the important role of this ectodermal layer in mutual adhesion and fusion of the neural folds at the midline [2, 43], we checked the expression of E-cadherin in our mutant mice (Fig. 7). In control embryos, the E-cadherin-positive non-neural ectoderm nicely overlays the tips of the neural folds (Figs. 7b,i), and forms a continuous lining as soon as the neural tube is closed (Fig. 7d,j), fully in line with the function of this tissue during neural fold contact and fusion. In contrast, in young mutant embryos (12–13 somites), the non-neural ectoderm, featured by E-cadherin expression, stops extending dorsally without covering the tip of the p120ctn-lacking neural fold (arrowhead in Fig. 7f; arrow in Fig. 7k). This defect is even more clear in older mutant embryos, where p120ctn-lacking mushroom structures are not at all covered by E-cadherin-positive cells (arrowhead in Fig. 7h).

### Beta-catenin expression in brains of p120ctn^fl/fl^;Wnt1Cre embryos

**Fig. 8:**
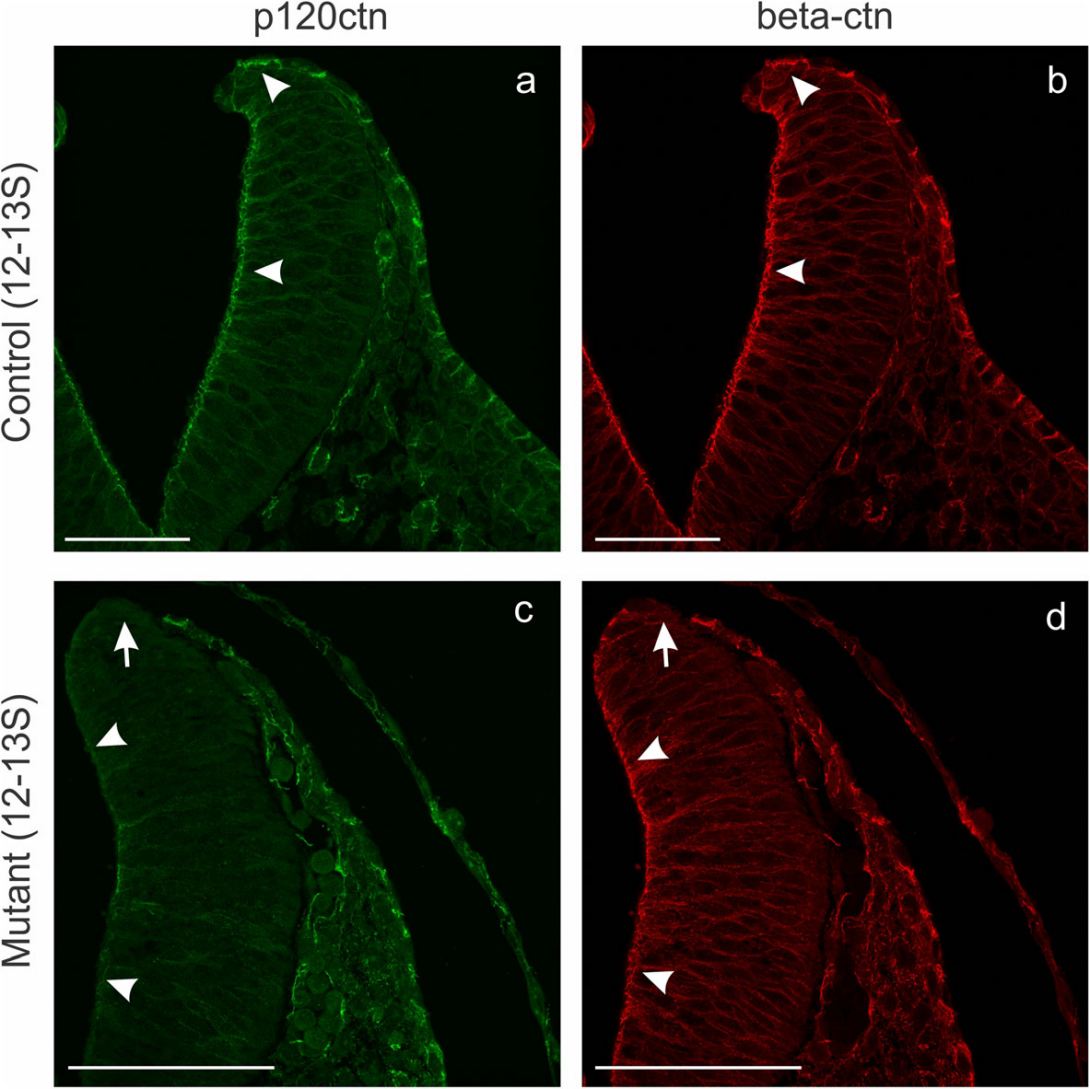
Focal abnormality of β-catenin expression in the neural folds of p120fl/fl;Wnt1Cre mutant mice. Immunofluorescence of p120ctn (**a**, **c**) and β-catenin (**b**, **d**) on neural folds in the hindbrain region of a control 12–13 somite embryo (**a**, **b**) and a mutant 12–13 somite embryo (**c**, **d**). In the control embryo, β-catenin was nicely coexpressed with p120ctn (arrowheads in **a**, **b**). In the mutant embryo, both p120ctn and β-catenin were lost at the very tip of the neural folds (arrows in **c**, **d**), but the p120ctn-lacking lateral side of the neural folds retained strong β-catenin positivity (arrowheads in **c**, **d**). Scale bars: 20 μm

The cytoplasmic C-terminal ends of all classic cadherins associate with β-catenin, while the more membrane-proximal domains of these cytoplasmic tails associate with p120ctn. Hence, expression of β-catenin at the plasma membrane reflects the presence of classic cadherins, including several with expression in the neural plate, neural folds or neural tube: N-cadherin, cadherin-6, cadherin-7 [44, 45]. At the 12–13 somite stage, β-catenin colocalized with p120ctn along the whole neural fold of control embryos (Fig. 8a,b arrowheads). Interestingly, when p120ctn was ablated in the neural folds of p120ctn^fl/fl^;Wnt1Cre embryos, this was not associated with decreased β-catenin expression (Fig. 8c,d; arrowheads), except for the very top of the folds (Fig. 8c,d; arrows). The difference in expression between the tips of control and mutant neural folds for both p120ctn and β-catenin reflects the focal absence of the overlying non-neural ectodermal layer in the mutants (see above). At a later stage (18-22S), the opened neural folds in the p120ctn^fl/fl^;Wnt1Cre embryo were negative for p120ctn, but still positive for β-catenin (Fig. S6d,e; notice the red color in the overlay of Fig. S6f). It is noteworthy that β-catenin was locally aggregated in these opened folds (Fig. S6e, arrows), what is in full accordance with the observations on N-cadherin (Figs. 6q and S5d).

### Expression of cortactin and Shroom-3 in brains of p120ctn^fl/fl^;Wnt1Cre embryos

**Fig. 9:**
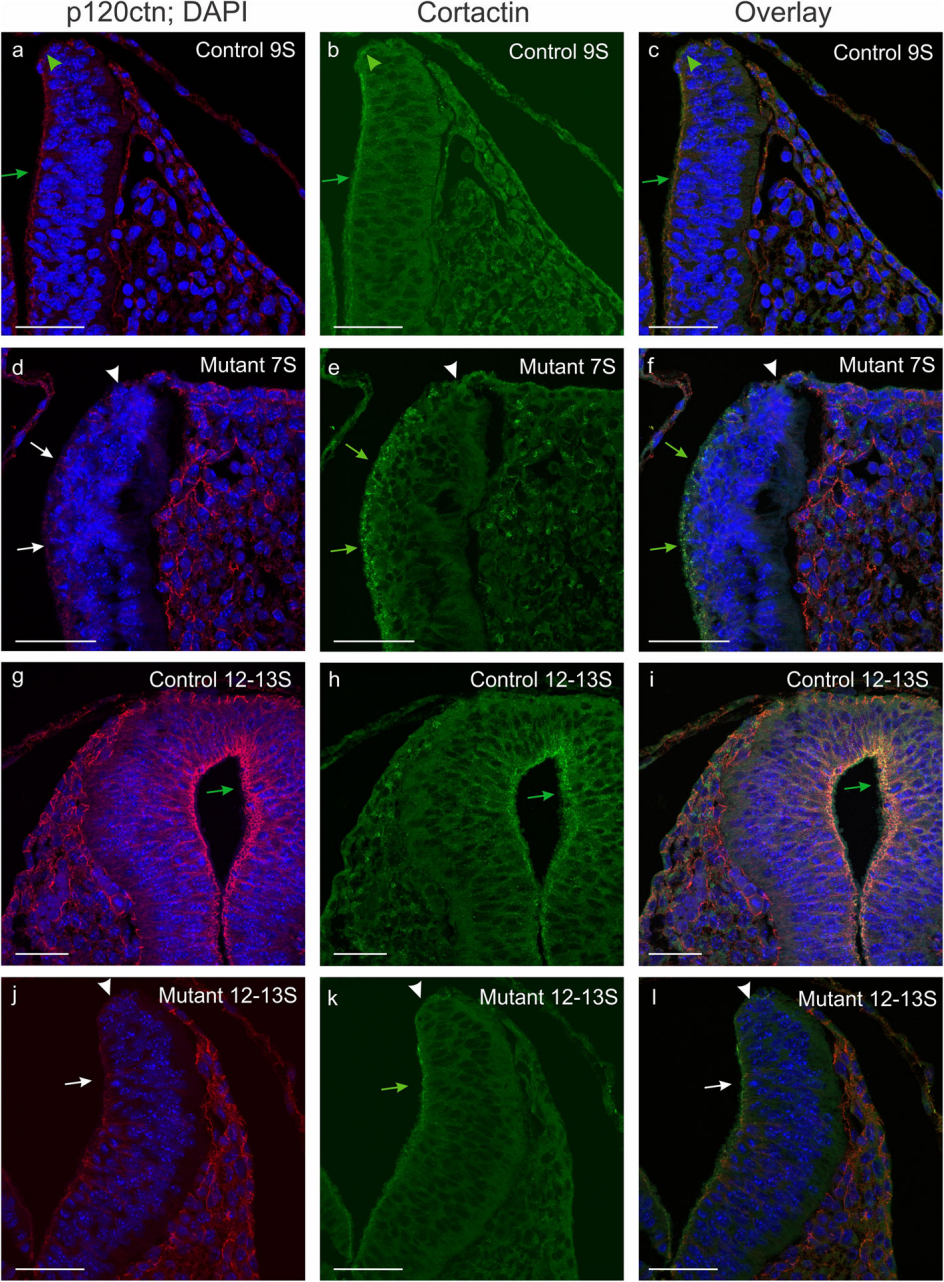
Focal abnormality of cortactin expression in the neural folds of p120fl/fl;Wnt1Cre mutant mice. **a**-**l** Immunofluorescence of p120ctn (left panels; nuclear counterstaining by DAPI) and cortactin (middle panels; overlay in right panels) on neural folds/tube in the hindbrain region of young embryos. Detection was on 9-somite (**a**-**c**) and 12–13 somite (**g**-**i**) control embryos, and on 7-somite (**d**-**f**) and 12–13 somite (**j**-**l**) mutant embryos. Green annotations point at positive signals; white annotations point at expression deficiency; arrows point at lateral sides of neural folds/tube; arrowheads point at the very tip of neural folds. In the control embryos (**a**-**c**, **g**-**i**), p120ctn and cortactin were coexpressed in neural folds/tube (arrows, arrowheads). In the mutant embryos (**d**-**f**, **j**-**l**), both p120ctn and cortactin were lost at the very tip of the neural folds (arrowheads), but the p120ctn-lacking lateral side of the neural folds retained cortactin positivity (arrows). Scale bars: 20 μm

Formation of membrane ruffles, lamellipodial extension, but also formation of cadherin-mediated cell-cell junctions were reported to be dependent on the molecular complex between cortactin and p120ctn [46]. Therefore, we analyzed the expression of cortactin in our control and mutant embryos (Fig. 9). In young 7-somite mutant embryos, we did not observe an obvious decrease in cortactin expression in neural folds with p120ctn ablation (Fig. 9d-f; arrows), as compared to control 9-somite embryos (Fig. 9a-c; arrows). In control embryos of 12–13 somites, the closed neural tube showed high co-expression of p120ctn and cortactin at the ventricular side of the neural tube (Fig. 9g-i; arrows). In p120ctn-lacking neural folds of 12-13S mutant embryos (Fig. 9j-l), the expression of cortactin was similar (arrow in Fig. 9k) to those of 9S control embryos (arrow in Fig. 9b), except for the very tip of the neural folds where defective p120ctn expression correlated with decreased cortactin expression (Fig. 9j-l; arrowheads). Although the latter folds were not closed, they were bended inwards in contrast to the folds of younger 7S mutant embryos (Fig. 9d-f). This indicates that a cortactin defect at the dorsolateral hinge points is unlikely to be a major cause of the NTDs observed in p120ctn^fl/fl^;Wnt1Cre embryos. On the other hand, a critical role of cortactin at the dorsal fusion point in the midline may be affected in our mutants.

**Fig. 10:**
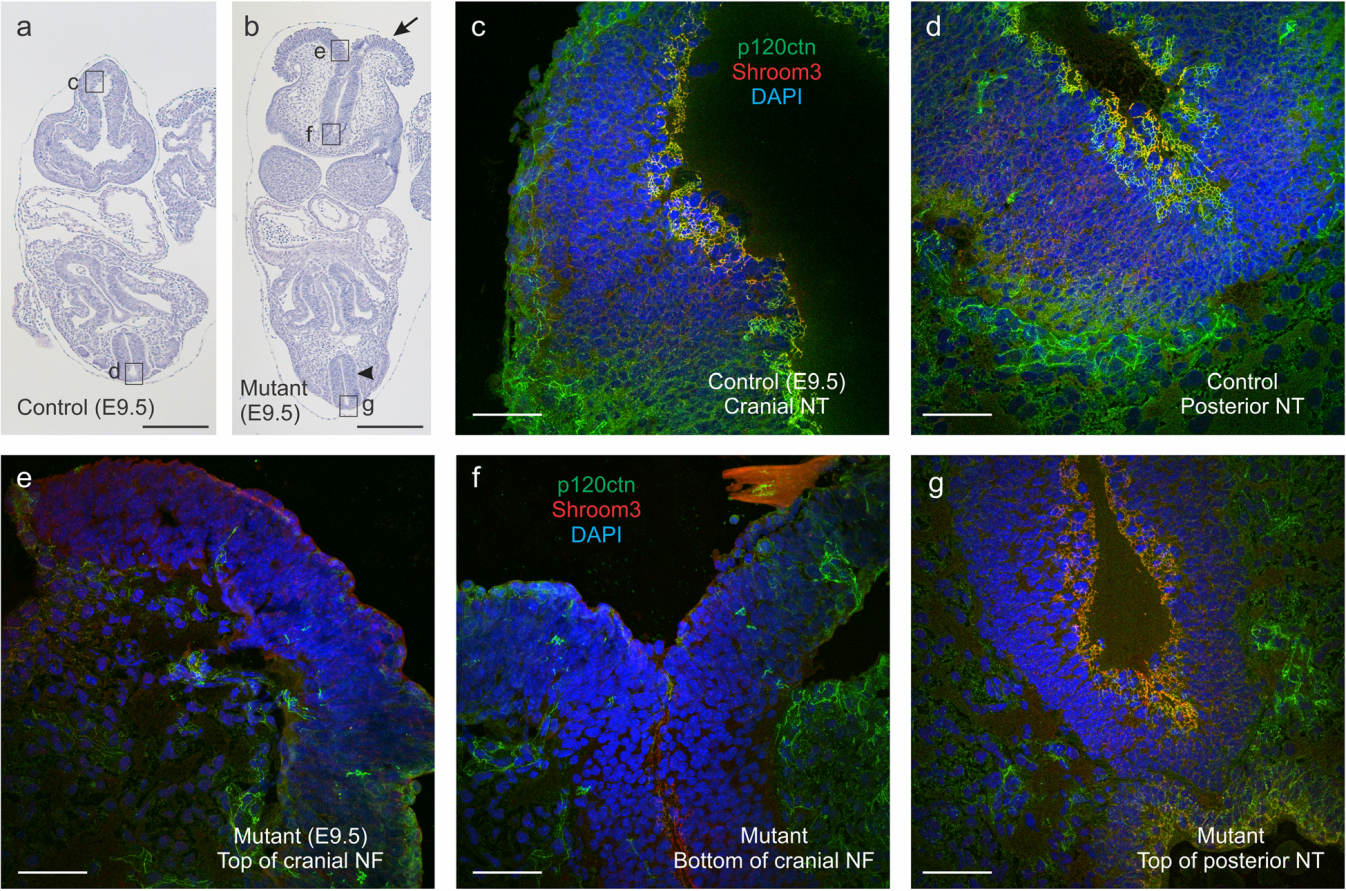
Deficiency of Shroom3 expression in the cranial neural folds of p120fl/fl;Wnt1Cre mutant mice. **a**, **b**. Coronal H&E stained whole-body sections were compared between a control E9.5 embryo (**a**) and a mutant E9.5 embryo (**b**). There is no closure for the cranial neural folds of the mutant embryo and NTD is obvious (**b**, arrow). In contrast, the caudal neural tube is normally closed (**b**, arrowhead). For areas comparable to those annotated by boxes **c**-**g** in **a** and **b**, confocal immunofluorescent analysis for p120ctn (green) and Shroom3 (red) (nuclear counterstaining by DAPI) was performed on specifically prepared samples (see Materials and Methods). In control samples (**c**, **d**) and in the posterior neural tube of the mutant (**g**), p120ctn and Shroom3 were nicely coexpressed, whereas in the cranial neural folds (**f**) and the NTD (**e)** of the mutant, p120ctn and Shroom3 were both ablated. Scale bars for **a**,**b**: 200 μm; for **c**-**g**: 20 μm

p120ctn has been shown to be necessary for apical junctional recruitment of Shroom3, and to interact genetically with Shroom3 with respect to NTD and ocular malformations [47]. We analyzed the expression patterns of p120ctn and Shroom3 by double immunofluorescence on cranial and posterior neural folds (Fig. 10). In control embryos p120ctn and Shroom3 were nicely co-expressed at intercellular contacts at the top of the ventricular side of the neural tube (Fig. 10a,c,d). In contrast, p120ctn-negative cranial neural folds of mutant embryos displayed loss of Shroom3 (Fig. 10b,e,f), whereas the closed neural tube at the posterior side of these embryos showed co-expression of p120ctn and Shroom3 (Fig. 10b,g), alike the situation in control embryos (Fig. 10a,d). This indicates that p120ctn loss in our mutant mice displaces the actin-binding protein Shroom3 from the apical side of cranial neural folds what is likely to contribute to the observed NTDs.

## Discussion

The armadillo family member p120ctn has many functions in cells [18, 48, 49], but its role in vivo remains largely unaddressed. Using a full p120ctn knock-out approach, we found that up to the early stage of gastrulation, mouse development occurs normally in the absence of p120ctn [29]. Gastrulation yields three germ layers, the E-cadherin positive epiblast-derived ectoderm and endoderm, and the newly emerging N-cadherin positive mesoderm. Remarkably, p120ctn is uniformly expressed in all three germ layers [50], and in absence of p120ctn a reduction of the corresponding cadherin in the respective germ layers was observed without affecting the cadherin switching itself [28]. Using a structure-function approach we showed that the stabilization of E-cadherin by p120ctn is crucial for primitive endoderm specification [29]. On the other hand, p120ctn dampens Wnt signaling and as such controls proper migration and proliferation of nascent mesoderm that emerges from the primitive streak [28]. In absence of p120ctn, Wnt activity is increased at the primitive streak and induces posterior axis duplication in mice [28]. This phenotype is in line with the formation of a secondary embryonic axis what is observed upon overexpression of Wnt signaling components in both vertebrate and invertebrate cleavage-stage embryos [51–54]. We could confirm this posterior axis duplication in our p120ctn-null embryos, although defects seen in neurulation were also very obvious. Since the posterior axis duplication could affect the neurulation defects and vice versa, we started to use Wnt1-Cre mice in order to restrict the p120ctn ablation to cells that give rise to the neural tube. The Wnt1-Cre system enables conditional deletion of target molecules in the midbrain, hindbrain and NC cells.

Neural tube formation and closure are among the most complex developmental events during embryonic development requiring several morphological changes that need to be tightly coordinated. Many cellular processes are involved, including cell shape change during neural plate formation, neural plate bending, ECM breakdown, formation of actin-rich protrusions at the leading edge, and specific cell-cell adhesion at the dorsal midline [6]. By using Wnt1-Cre mice several components of the cadherin-catenin complex have previously been shown to be involved in mouse brain and neural tube formation. Luo et al. demonstrated that Wnt1-driven ablation of N-cadherin results in exencephaly and embryonic death [55]. Furthermore, like the EMT process during neurulation, the formation of mesoderm and NC cells require regulated loss of cell–cell adhesion during normal embryonic development [26, 56, 57]. Brault et al. reported that inactivation of the β-catenin encoding gene in Wnt1-expressing cells resulted in serious brain malformations and failure of craniofacial development [58]. By using Wnt1-Cre-mediated deletion of p120ctn, we demonstrated here that p120ctn plays an important role in mouse craniofacial and central nervous system development. In 30% of the animals with site-specific ablation, NTDs with unfused neural folds in the anterior region were generated, evolving over time to prominent exencephaly with an abnormally large protruding mid- and hindbrain and a missing skull vault. While the skull bone defects could be secondary to the brain defects, the observed exencephaly was likely a direct result from the essential role of p120ctn in neurulation.

During normal cranial neurulation, several phases have been discerned [2, 4]: i. a lifting phase, featured by the generation of a median hinge point (MHP) in the neural plate producing bi-convex neural folds bulging outwards, ii. A bending phase, featured by the formation of dorsolateral hinge points (DLHP) at the edges of neural folds, so that the flipped neural folds approach the midline with bi-concave morphology, and iii. The fusion phase, when the tips of the bended neural folds touch each other and fuse in the midline, forming a neural tube. On the basis of many analyzed p120ctn^fl/fl^;Wnt1Cre mutant embryos, we found no evidence for abnormalities in the lifting phase of p120ctn-lacking neural folds. Moreover, the hindbrains of our mutant embryos of 12–13 somites showed consistently a inwards bended morphology indicating the generation of a DLHP (for instance, Figs. 6g,h; 7e,f; 8c,d; 9j-l), while this is apparently not yet the case for younger embryos of 7–10 somites (for instance Figs. 6c,d; 9d-f).

Specifically the mid- and hindbrain regions were affected in our mutant mice with ablated p120ctn, which is in line with the expression of Wnt1 in the isthmus where it controls the regional patterning of the mid- and hindbrain [59]. The mechanism underlying the observed exencephaly may be similar to the one causing unpolarized epithelial tumor-like cell masses in the lumen of salivary ducts after conditional knockout of p120ctn in the salivary gland in mice [11]. In the latter model, downregulation of the tumor suppressor E-cadherin has been proposed as a mechanism for the observed phenotype. This E-cadherin drop should trigger the nuclear and oncogenic translocation of β-catenin, and the alleviation of the repressive activity of Kaiso [16, 17, 60]. The following findings argue against such an mechanism in our model. Anatomically, the brain malformations in our p120ctn mutants are clearly different from those in mice with Wnt1-Cre driven β-catenin ablation [58]. In the β-catenin mutants entire parts of mid- and hindbrain are missing, as demonstrated by the analysis of brain markers *Otx2*, *En1* and *Fgf8*, and which is likely due to defects in the Wnt signaling pathway [58]. In our p120ctn^fl/fl^;Wnt1Cre mutants all brain parts were present although deformed. We also checked whether p120ctn ablation affects genes important for brain development including isthmus specification, by analyzing several midbrain and hindbrain markers by in situ hybridization. None of the tested brain markers (*Wnt1*, *Otx2*, *En1* and *Fgf8*) showed obvious expression differences in either location or quantity between control mice and mutant embryos displaying exencephaly (Fig. 4). Moreover, we found no evidence for nuclear translocation of β-catenin in p120-lacking neural folds (Figs. 8 and S6). We also checked for abnormally high proliferation activity in p120ctn lacking neural folds by staining for phospho-Histone 3. We found no convincing evidence for such defects (Figs. 5 and S4). Therefore, the brain malformations in p120ctn mutants are unlikely to be due to disruption of p120ctn regulation of target gene transcription, leading for instance to activation of oncogenic β-catenin.

On the other hand, in view of the known biological activities of p120ctn, the brain defects in our p120ctn^fl/fl^;Wnt1Cre mice could be explained by defective functionality of cadherin-mediated cell-cell adhesion. Cadherins such as E-cadherin and N-cadherin and their mutual switching have been studied in detail during central nervous system development [25, 61]. The NTDs and exencephaly caused by Wnt1-Cre mediated deletion of N-cadherin in mouse [55], resemble the phenotype that we observed in p120ctn mutant mice. Also, it has been reported that p120ctn deletion in the forebrain by the Emx1-Cre system leads to reduced levels of N-cadherin in the brain and consequently to developmental defects [30]. It is therefore somewhat surprising that in our study N-cadherin expression was not dramatically affected in the plasma membrane of p120ctn-ablated brain cells. The same was true for β-catenin, which associates with the cytoplasmic domains of classic cadherins including N-cadherin. Although mechanistically not well understood, the focal loss of both N-cadherin and β-catenin at the very top of the inward bending elevated neural folds in 10–13 somite embryos (Figs. 6h and 8d) seems to be important. It is exactly at this specific location that the neural inter-fold contact and subsequent fusion in the midline is to be expected.

Midline fusion of neural folds is initiated by lamellipodial cell protrusions from the apposing neural fold apices, which then start to interdigitate [2]. Surface non-neural ectoderm, expressing E-cadherin, and neuroepithelial cells, expressing N-cadherin, will finally be remodeled at the interfold contacts, yielding at the midline separate continuous cell sheets of ectodermal and neuroepithelial type. The non-neural surface ectodermal cells located at the neural fold edge were reported to be different morphologically and dynamically from both the underlying neuroectoderm and the adjacent non-neural surface ectoderm, and are considered mainly responsible for the initial contact, the zipping and fusion of juxtaposed cranial neural folds [43, 62]. It is thus most interesting that in our p120ctn^fl/fl^;Wnt1Cre embryos E-cadherin is focally lost in these non-neural surface ectodermal cells (Fig. 7). This may be the major molecular cause for the observed NTDs. It implies an intriguing inductive negative interaction of neuroepithelial cells with ablated p120ctn on the ectodermal cells with WT p120ctn at this site. We indeed found no evidence for loss of p120ctn in the non-neural surface ectodermal cells in our mutant mice.

It is also relevant that, at least in non-neural cells, p120ctn has been shown to regulate the actin cytoskeleton and lamellipodial dynamics by modulating the activity of RhoGTPases [19, 48], by cooperating with cortactin, an actin- and Arp2/3 complex-binding protein [46], and by interacting with Shroom3 [47]. Shroom3 has been shown to be critical for cranial neural tube closure [63], as it mediates recruitment of Rho kinases to apical cell junctions what leads to apical constriction [64, 65]. Apical constriction is most important for neural fold bending along the body axis [6]. We believe therefore that the following observations are at the basis of the defective cranial neural tube closure in a significant fraction of p120ctn^fl/fl^;Wnt1Cre embryos. Apparently, the Wnt1Cre-induced p120ctn ablation leads to a focal loss of N-cadherin and cortactin in the neuroepithelium at the very tip of the neural fold (Figs. 6 and 9), and also at a loss of the E-cadherin-expressing non-neural surface ectodermal cells at this tip (Fig. 7). The combination of these abnormalities will result in failing neural fold contact at the midline, resulting in loss of neural fold fusion and eventually in the open folded structure at the origin of exencephaly. Although for technical reasons we could not check this at earlier developmental stages, the observed prominent loss of Shroom3 expression at the apical surface of cranial E9.5 neural folds in case of p120cn ablation (Fig. 10), may contribute to the open fold structure when the fusion at the midline fails.

## Conclusions

Our results collectively indicate that p120ctn is strictly required for normal neurogenesis and neurulation. In full p120ctn KO mice, development was apparently normal until early gastrulation. Thereafter several defects in each of the three germ layers became apparent. We then focused on the role of p120ctn in neurulation by applying Wnt1-Cre driven ablation: 30% of the mutant embryos displayed exencephaly and died before birth. This number may be increased by testing different genetic backgrounds. It was puzzling that convincing p120ctn ablation in the lifted and bended neural folds did not consistently result in loss of N-cadherin or β-catenin. In contrast, the observed deficient neural fold fusion could be correlated with focal losses of cell adhesion components (N-cadherin, β-catenin) and actomyosin complexes (cortactin) at the very apical side of neural folds. Also, expression of the actin-binding protein Shroom 3 was affected. Another relevant observation was the focal loss of E-cadherin-expressing surface non-neural ectoderm at neural fold tips with p120ctn ablation. This interesting genetic model of NTD is readily available and the further combination with other Cre mice or with mouse mutants of other key genes may reveal most interesting aspects of brain and skull formation.

## Methods

### Generation of full and conditional p120ctn-knockout mice

Floxed p120ctn (p120ctn^fl/fl^) mice [11] were crossed with, respectively, Nestin-Cre (allowing Cre expression in all adult organs) [66], or Wnt1-Cre transgenic mice [33]. Breeding and genotyping of mutant p120ctn mice was described before [29]. Timed matings were performed to obtain embryos at specific stages [67]. Somites were counted during dissection under a stereo microscope. We genotyped embryos by PCR on genomic DNA isolated from the yolk sac. All animal experiments were approved by the Experimental Animal Ethics Committee of Ghent University. Altogether, for the p120ctn^fl/fl^;Wnt1Cre genotype we analyzed more than 300 embryos (from E8.5 to E18.5). About 100 of them were control (p120ctn^fl/fl^;Wnt1Cre^**−**^) and about 200 of them were knockout mice (KO: p120ctn^fl/fl^;Wnt1Cre^+^).

All mice were bred and housed at the Vlaams Instituut voor Biotechnologie (VIB, Ghent University) in a specific-pathogen free facility. Mouse experiments were performed in accordance with the Ethics Committee of the Faculty of Science of Ghent University, and were meeting the requirements of the European Directive 2010/63/EU. All sections of this report adhere to the ARRIVE Guidelines for reporting animal research [68]. Adult and young mice were killed by cervical dislocation. Embryos and newborn mice were killed by decapitation. A completed ARRIVE guidelines checklist is included as Additional file 7. The Animal Facility Procedures and Licenses of the Center for Inflammation Research (Ghent University and VIB, Ghent, Belgium) are overviewed in Additional file 8.

### Histology and molecular analysis of p120ctn^fl/fl^;Wnt1Cre mice

Embryos were collected in phosphate buffered saline (PBS) and fixed overnight in 4% paraformaldehyde at room temperature. They were dehydrated gradually and embedded in paraffin for sectioning. Sections were used for hematoxylin and eosin (H&E) staining, toluidine blue staining or immunostaining. Whole-mount in situ hybridization was performed following a standard protocol [69]. Digoxigenin-labeled antisense riboprobes for *Wnt1*, *Otx2*, *En1* and *Fgf8* were used. Plasmids for the riboprobes were a kind gift from D. Huylebroeck (K.U. Leuven and VIB, Belgium). X-gal staining was performed as described [70].

### Immunofluorescence (IF) and immunohistochemistry (IHC)

To unmask antigens, paraffin sections were rehydrated and treated with citrate buffer in a Retriever apparatus (PickCell Laboratories, Amsterdam, The Netherlands). The sections were incubated with primary antibodies at 4 °C overnight. Primary antibodies used for IHC on total p120 KO embryos were: mouse-anti-p120ctn (BD Transduction Laboratories, San Jose, CA, USA; dilution 1:200), mouse-anti-N-cadherin (Zymed, South San Francisco, CA, USA; 1:500), mouse monoclonal anti-Nestin (BD Transduction Laboratories; 1:1000), rabbit polyclonal anti-βIII-tubulin (BD Transduction Laboratories; 1:3000), mouse monoclonal anti-smooth muscle actin (SMA; Sigma; 1:100), mouse monoclonal anti-cardiac isoform of Troponin-T (NeoMarkers, Fremont, CA; 1:100). Primary antibodies for the analysis of the p120ctn^fl/fl^;Wnt1Cre embryos were: rabbit monoclonal anti-delta1 catenin (Abcam, Cambridge, UK; dilution 1:300), mouse monoclonal anti N-cadherin (Zymed; 1:300), mouse monoclonal anti-E-cadherin (BD Transduction Laboratories; 1:500), mouse monoclonal anti-β-catenin (BD Transduction Laboratories; 1:400), rabbit polyclonal anti-cortactin (Santa-Cruz Biotechnology, Heidelberg, Germany; 1:200), mouse monoclonal anti-p120ctn (BD Transduction Laboratories; 1:300) and rabbit polyclonal anti-phospho-Histone H3 (Bethyl Laboratories, BIOKÉ, Leiden, The Netherlands; 1:500). Affinity-purified rabbit anti-Shroom3 (1:400) was kindly provided by Prof. M. Takeichi [64].

For IF, slides were labeled with goat anti-rabbit IgG (H + L) secondary antibody DyLight488 (dilution 1:1000) or with goat anti-mouse IgG (H + L) cross-adsorbed secondary antibody Alexa Fluor 568 (1:500), both from Thermo Fisher Scientific. Counterstaining was performed with Hoechst and all slides were mounted with 1% propyl gallate in glycerol before analysis with a Leica Sp5 confocal scanning microscope. For IHC, the sections were incubated with secondary antibodies conjugated with biotin (Dako, Heverlee, Belgium). Avidin-biotin complexes were made (Vector Laboratories, Burlingame, CA, USA) and the signal was detected with diaminobenzidine (Dako). For Shroom3 detection, mouse embryos were frozen in OCT compound immediately after dissection. Sections of 10 μm were made with a cryostat and dried for 1 h at room temperature. The slides were then fixed with methanol at − 20 °C for 10 min. Thereafter, we immediately performed staining by three washing steps in PBS, blocking in 3% goat serum, 1% BSA, followed by overnight incubation with anti-Shroom3 antibody at 4 °C.

### Neurogenesis in mouse embryonic stem cells (mESCs)

Culturing mESCs was described previously [29, 71]. N2B27 medium was prepared as described [72]. For 2i + LIF medium, 1 μM PD0325901 (Axon Medchem, Groningen, NL), 3 μM CHIR99021 (Axon Medchem), and 1000 U LIF/ml (Millipore) were added to N2B27 medium. A protocol for directed neural differentiation was reported before [32]. In brief, 3 × 10^6^ naive mESCs were plated on day 0 in a 10-cm bacterial petri dish to form embryoid bodies (EBs) in FBS-containing medium. On day 2 the EB medium was refreshed; on day 4 it was changed to N2B27 + retinoic acid (Sigma-Aldrich, R2625, 500 nM), and this was refreshed on day 6. Between day 8 and day 15, EBs were cultured in N2B27, which was refreshed every other day. EBs were then fixed, embedded in paraffin, sectioned and processed for IF.

### Teratoma formation

Control and p120ctn-null mESCs were trypsinized and 1.5 × 10^6^ cells were injected subcutaneously into the flank of immunocompromised nude mice. Growing teratomas were isolated 5 to 8 weeks later and processed for IHC analysis.

## Supplementary information

**Additional file 1: Figure S1.** Normal mesoderm and cardiomyocyte formation upon p120ctn loss in mouse. IHC analysis of mesodermal marker SMA (**a**) and of cardiac Troponin-T (**b**) on paraffin sections from E9.5 control and p120ctn-null embryos. Lower panels are magnifications of identical or consecutive sections as depicted in the upper panels. Arrows point at unfused neural folds. Scale bars: 100 μm.

**Additional file 2: Figure S2.** IHC for Nestin and βIII-tubulin on sections of teratomas that were derived from subcutaneous injection in athymic nude mice of control or p120ctn-null mESCs. Scale bars: 100 μm.

**Additional file 3: Figure S3.** Whole mount X-gal staining of young Wnt1-Cre/R26R mouse embryos (6 to 17 somites [S]; E8.0 – E9.0) revealed the spatiotemporal expression characteristics of Wnt1-Cre. Lateral views (**a, b, c, e, f**) and a dorsal view (**d**) of the embryos are shown.

**Additional file 4: Figure S4.** No prominent cell proliferation changes in p120^fl/fl^;Wnt1Cre mutant mice. Sections through the hindbrain region of 18–22 somite embryos are shown. **a-c.** Expression of p120ctn was prominent in the neural tube of a control embryo (**a**), but ablated in the closed neural tube of one mutant embryo (**b**; arrow), and in the NTD of another mutant embryo (**c**; arrow). **d-f**, Immunodetection of phosphorylated Histone 3 (pH 3) showed similar activity in control and mutant embryos. Scale bars: 20 μm.

**Additional file 5: Figure S5.** Organizational abnormalities of N-cadherin expression in the cranial neural folds and NTD of p120fl/fl;Wnt1Cre mutant mice. IHC of p120ctn (**a**) and of N-cadherin (**c**) on a cranial neural tube of an E9.5 control embryo showed prominent coexpression. In an E9.5 (25–30 somites) mutant embryo, p120ctn was strongly ablated (**b**; asterisk, section artefact), whereas strong expression of N-cadherin was retained (**d**). In the latter situation, focal N-cadherin aggregation and a non-coherent exposed cell layer were visible (**d**, arrows), in contrast to the uniform N-cadherin expression and the closed cell layer facing the ventricular lumen in the control neural tube (**c**, arrowheads). Scale bar: 20 μm.

**Additional file 6: Figure S6.** Organizational abnormality of β-catenin expression in the neural folds and NTD of 18–22 somite p120fl/fl;Wnt1Cre mutant mice. Immunofluorescence of p120ctn (**a, d**) and β-catenin (**b, e**; overlay in **c, f**) in the cranial neural tube of a control 18–22 somite embryo (**a-c**), or in the cranial neural folds/NTD of a mutant 18–22 somite embryo with NTD (**d-f**). In the control embryo, β-catenin was nicely coexpressed with p120ctn. In the mutant embryo, p120ctn was lost in the NTD (**d**), but β-catenin was still expressed, although focally with aggregated appearance (**e**, arrows). Scale bar: 10 μm.

**Additional file 7.** Completed ARRIVE Guidelines Checklist.

**Additional file 8.** Animal Facility Procedures and Licenses of the Center for Inflammation Research, Ghent University and VIB, Ghent, Belgium.

## Data Availability

All data generated or analysed during this study are included in this published article [and its supplementary information files].

## Abbreviations

DLHP: DorsoLateral Hinge Point
EBs: Embryoid Bodies
EMT: Epithelial-to-Mesenchymal Transition
H&E: Hematoxylin and Eosin
IF: ImmunoFluorescence
IHC: ImmunoHistoChemistry
KO: Knockout
mESCs: Mouse Embryonic Stem Cells
MHP: Median Hinge Point
NC: Neural Crest
NTD: Neural Tube Defect
p120ctn: p120 catenin
PBS: Phosphate buffered saline
pH 3: Phopho-Histone 3
SMA: Smooth Muscle Actin
