# Supplementary figures and images for: Neural defects caused by total and Wnt1-Cre mediated ablation of p120ctn in mice
Source: BMC Dev Biol. 2020 Aug 3;20:17. doi: 10.1186/s12861-020-00222-4 (PMC7398255; doi:10.1186/s12861-020-00222-4)

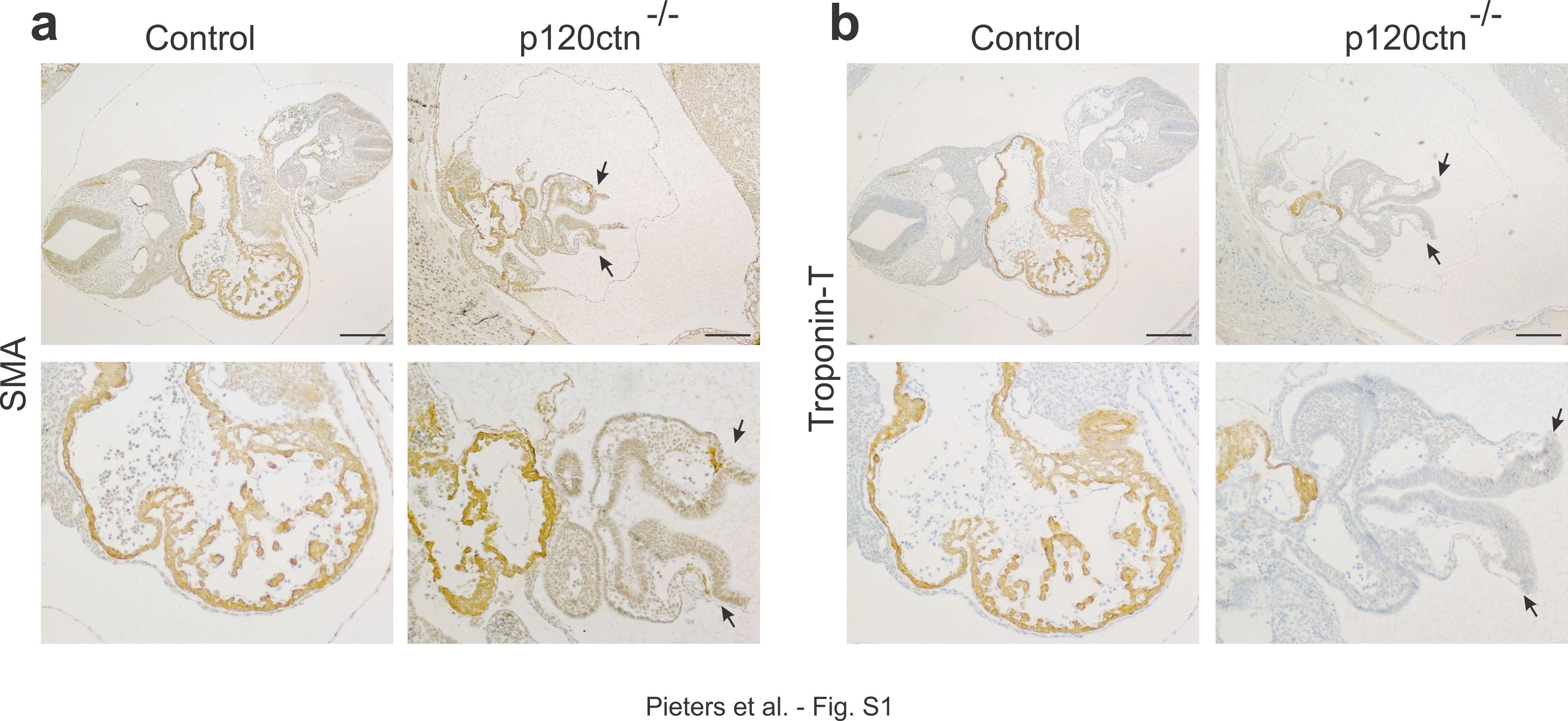

Supplement: Supplementary file 1 — Additional file 1: Figure S1. Normal mesoderm and cardiomyocyte formation upon p120ctn loss in mouse. IHC analysis of mesodermal marker SMA (a) and of cardiac Troponin-T (b) on paraffin sections from E9.5 control and p120ctn-null embryos. Lower panels are magnifications of identical or consecutive sections as depicted in the upper panels. Arrows point at unfused neural folds. Scale bars: 100 μm. [file 12861_2020_222_MOESM1_ESM.jpg]

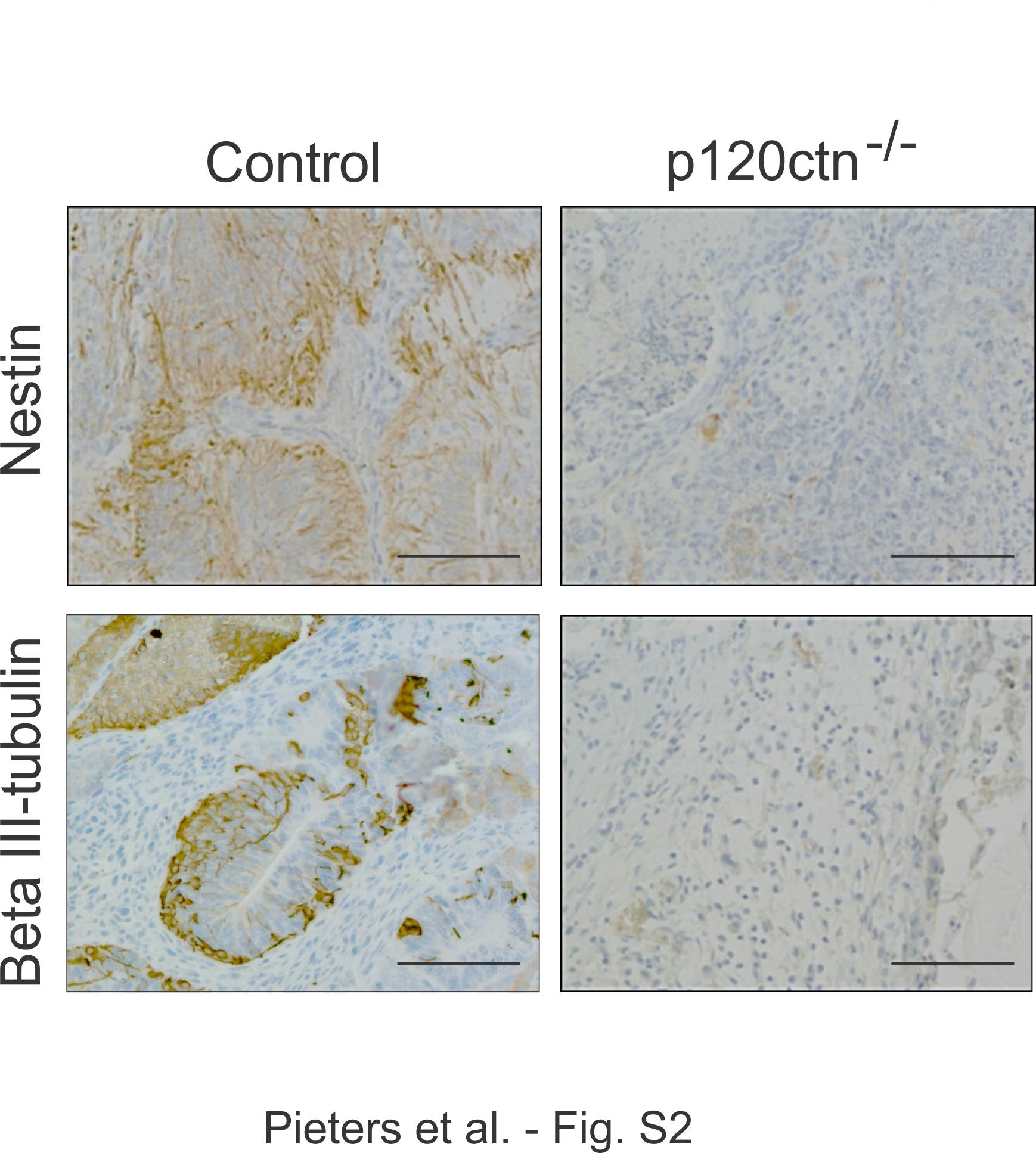

Supplement: Supplementary file 2 — Additional file 2: Figure S2. IHC for Nestin and βIII-tubulin on sections of teratomas that were derived from subcutaneous injection in athymic nude mice of control or p120ctn-null mESCs. Scale bars: 100 μm. [file 12861_2020_222_MOESM2_ESM.jpg]

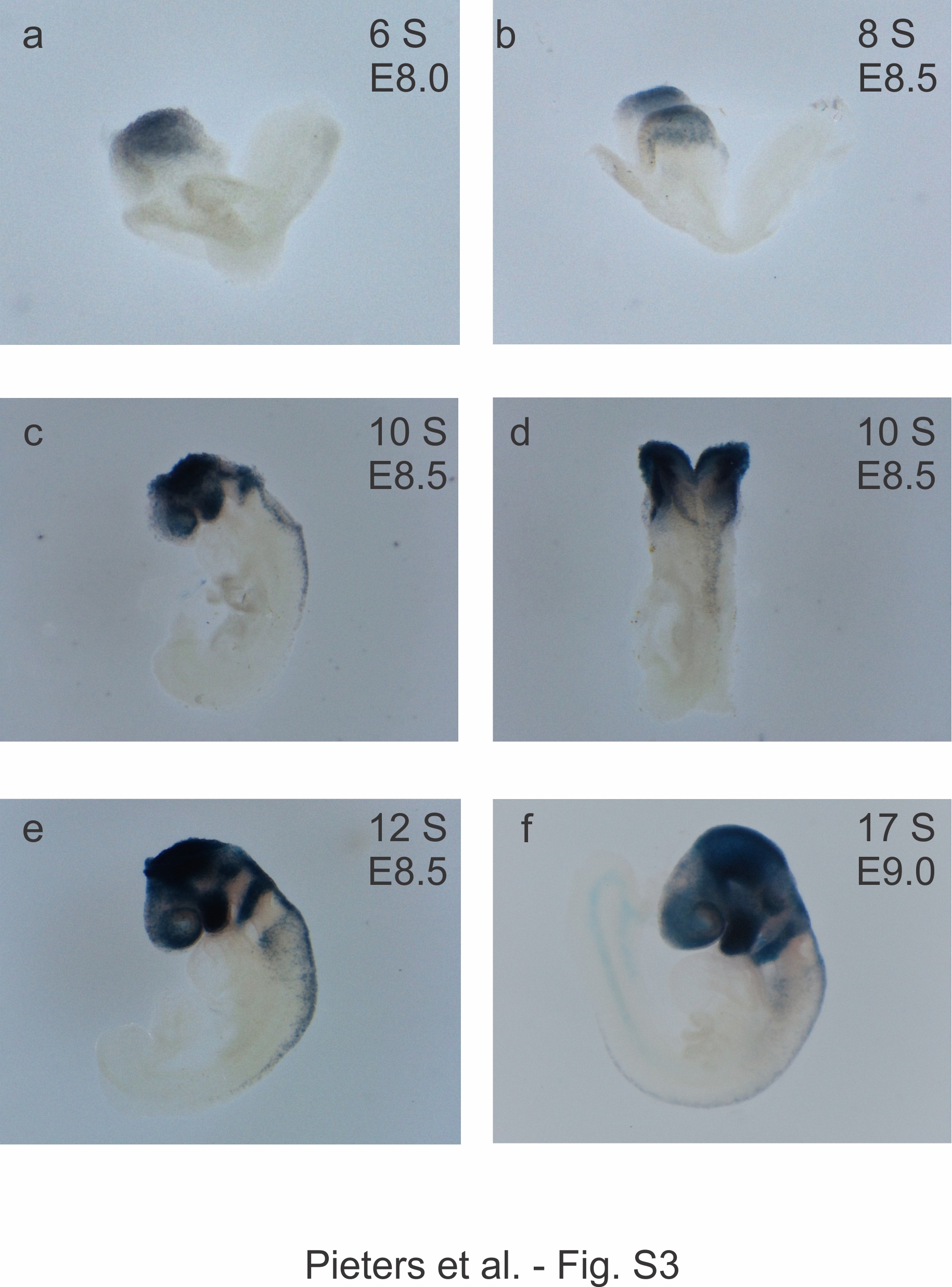

Supplement: Supplementary file 3 — Additional file 3: Figure S3. Whole mount X-gal staining of young Wnt1-Cre/R26R mouse embryos (6 to 17 somites [S]; E8.0 – E9.0) revealed the spatiotemporal expression characteristics of Wnt1-Cre. Lateral views (a, b, c, e, f) and a dorsal view (d) of the embryos are shown. [file 12861_2020_222_MOESM3_ESM.jpg]

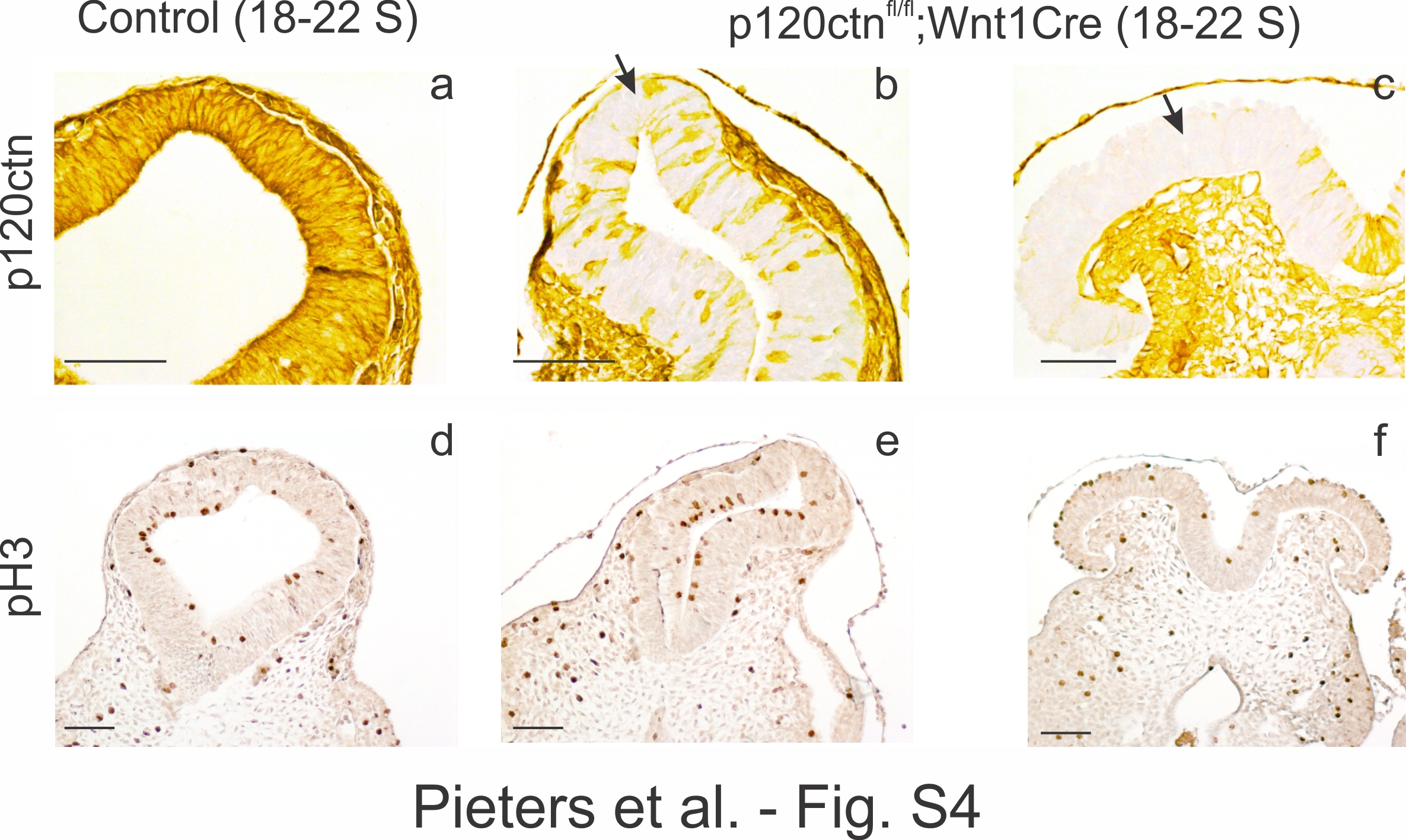

Supplement: Supplementary file 4 — Additional file 4: Figure S4. No prominent cell proliferation changes in p120fl/fl;Wnt1Cre mutant mice. Sections through the hindbrain region of 18–22 somite embryos are shown. a-c. Expression of p120ctn was prominent in the neural tube of a control embryo (a), but ablated in the closed neural tube of one mutant embryo (b; arrow), and in the NTD of another mutant embryo (c; arrow). d-f, Immunodetection of phosphorylated Histone 3 (pH 3) showed similar activity in control and mutant embryos. Scale bars: 20 μm. [file 12861_2020_222_MOESM4_ESM.jpg]

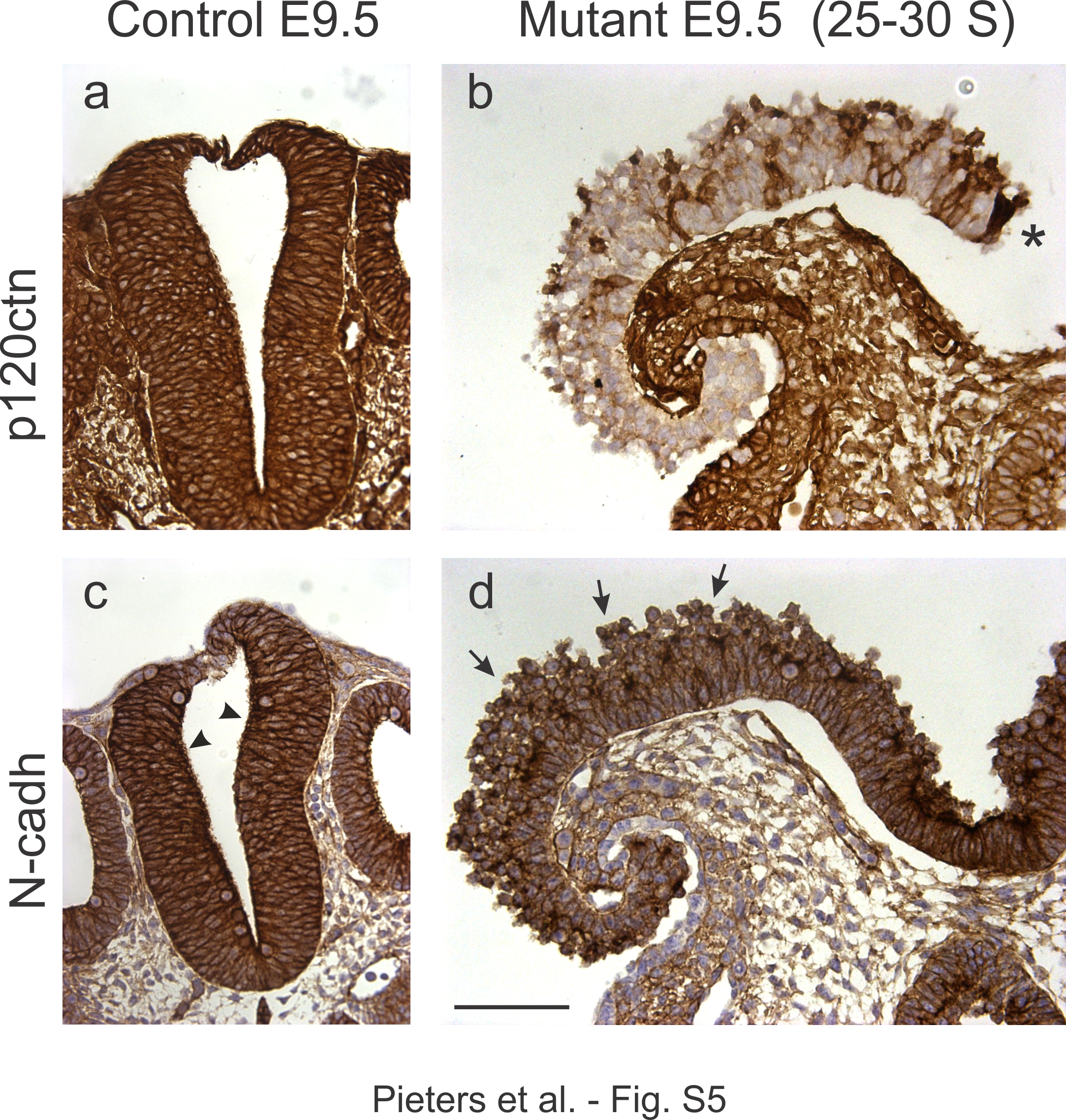

Supplement: Supplementary file 5 — Additional file 5: Figure S5. Organizational abnormalities of N-cadherin expression in the cranial neural folds and NTD of p120fl/fl;Wnt1Cre mutant mice. IHC of p120ctn (a) and of N-cadherin (c) on a cranial neural tube of an E9.5 control embryo showed prominent coexpression. In an E9.5 (25–30 somites) mutant embryo, p120ctn was strongly ablated (b; asterisk, section artefact), whereas strong expression of N-cadherin was retained (d). In the latter situation, focal N-cadherin aggregation and a non-coherent exposed cell layer were visible (d, arrows), in contrast to the uniform N-cadherin expression and the closed cell layer facing the ventricular lumen in the control neural tube (c, arrowheads). Scale bar: 20 μm. [file 12861_2020_222_MOESM5_ESM.jpg]

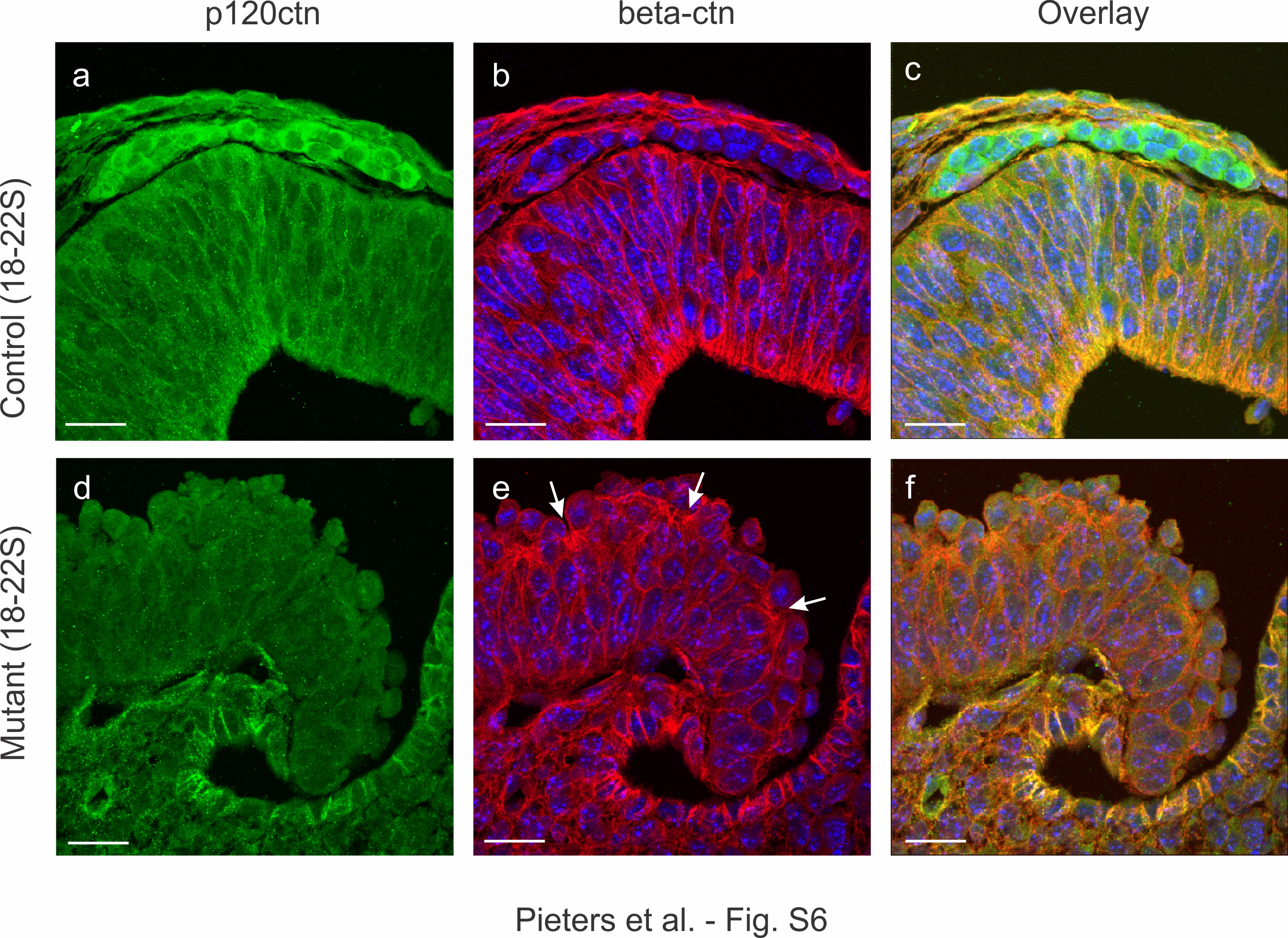

Supplement: Supplementary file 6 — Additional file 6: Figure S6. Organizational abnormality of β-catenin expression in the neural folds and NTD of 18–22 somite p120fl/fl;Wnt1Cre mutant mice. Immunofluorescence of p120ctn (a, d) and β-catenin (b, e; overlay in c, f) in the cranial neural tube of a control 18–22 somite embryo (a-c), or in the cranial neural folds/NTD of a mutant 18–22 somite embryo with NTD (d-f). In the control embryo, β-catenin was nicely coexpressed with p120ctn. In the mutant embryo, p120ctn was lost in the NTD (d), but β-catenin was still expressed, although focally with aggregated appearance (e, arrows). Scale bar: 10 μm. [file 12861_2020_222_MOESM6_ESM.jpg]
